# Midline Signalling Systems Direct the Formation of a Neural Map by Dendritic Targeting in the *Drosophila* Motor System

**DOI:** 10.1371/journal.pbio.1000200

**Published:** 2009-09-22

**Authors:** Alex Mauss, Marco Tripodi, Jan Felix Evers, Matthias Landgraf

**Affiliations:** 1 Department of Zoology, University of Cambridge, Cambridge, United Kingdom; 2 Friedrich Miescher Institut and Biozentrum, Department of Cell Biology, University of Basel, Basel, Switzerland; University of Washington, United States of America

## Abstract

A fundamental strategy for organising connections in the nervous system is the formation of neural maps. Map formation has been most intensively studied in sensory systems where the central arrangement of axon terminals reflects the distribution of sensory neuron cell bodies in the periphery or the sensory modality. This straightforward link between anatomy and function has facilitated tremendous progress in identifying cellular and molecular mechanisms that underpin map development. Much less is known about the way in which networks that underlie locomotion are organised. We recently showed that in the *Drosophila* embryo, dendrites of motorneurons form a neural map, being arranged topographically in the antero-posterior axis to represent the distribution of their target muscles in the periphery. However, the way in which a dendritic myotopic map forms has not been resolved and whether postsynaptic dendrites are involved in establishing sets of connections has been relatively little explored. In this study, we show that motorneurons also form a myotopic map in a second neuropile axis, with respect to the ventral midline, and they achieve this by targeting their dendrites to distinct medio-lateral territories. We demonstrate that this map is “hard-wired”; that is, it forms in the absence of excitatory synaptic inputs or when presynaptic terminals have been displaced. We show that the midline signalling systems Slit/Robo and Netrin/Frazzled are the main molecular mechanisms that underlie dendritic targeting with respect to the midline. Robo and Frazzled are required cell-autonomously in motorneurons and the balance of their opposite actions determines the dendritic target territory. A quantitative analysis shows that dendritic morphology emerges as guidance cue receptors determine the distribution of the available dendrites, whose total length and branching frequency are specified by other cell intrinsic programmes. Our results suggest that the formation of dendritic myotopic maps in response to midline guidance cues may be a conserved strategy for organising connections in motor systems. We further propose that sets of connections may be specified, at least to a degree, by global patterning systems that deliver pre- and postsynaptic partner terminals to common “meeting regions.”

## Abstract 2

During embryonic development of the motor system of *Drosophila*, motorneurons target their dendrites to different regions along the body axis in response to midline guidance cues.

## Author Summary

How neural networks governing locomotion are organised is less well understood than those governing sensory systems. In the *Drosophila* embryo dendrites form the input structures of motorneurons, and are arranged along the anterior-posterior axis in the central nervous system to reflect the distribution of body wall muscles in the periphery. Here we examine how a motorneuron dendritic map develops. We find that motorneurons target their dendrites also to distinct medio-lateral territories. This map appears to be “hard-wired” in that its formation does not require synaptic input or the proper positioning of partner terminals. Instead, dendritic targeting is determined by the responsiveness of individual motorneurons to midline guidance cues, mediated by the Slit receptor Robo and the Netrin receptor Frazzled. These findings complement and mirror similar results by others on the positioning of presynaptic axon terminals, and together they suggest a central role for global guidance cues in generating connectivity by delivering partner terminals independently of one another to common “meeting regions.”

## Introduction

Understanding the organisational logic of a neuronal network is a necessary step towards unravelling the mechanisms that underlie its specification and assembly. For many sensory systems, axon terminals are arranged in the central nervous system (CNS) to form neural representations of the topography or modality of the sensory neurons in the periphery [1]. This straightforward link between neuronal anatomy and function has fuelled the remarkable progress in identifying the underlying cellular and molecular mechanisms. In the visual system, for example, retino-topic connections are specified by matching gradients of axon guidance molecules in the retina and its target, the tectum/superior colliculus (for review see [2]). For motor systems in contrast, much less is known. A central organisational principle that we recently discovered in the *Drosophila* embryonic nerve cord is that the input structures of motorneurons, the dendrites, are distributed in the antero-posterior axis so that they form a neural “myotopic” representation of the body wall musculature in the periphery [3]. In vertebrates motor pool–specific differences of dendrite distributions have also been observed [4]–[6], suggesting that myotopic dendritic maps may constitute a conserved organisational framework for motor systems. Other manifest regularities of vertebrate motor systems, such as the grouping of motorneuron cell bodies into pools and columns, are thought to reflect primarily ontogenetic rather than functional relationships [7],[8]. The idea of myotopic maps implies that different dendritic territories represent, at least to a degree, different patterns of connectivity with presynaptic neurons. Support for this notion has been found in the mouse spinal cord where the expression of the transcription factor Pea3 in certain motor pools correlates with a particular dendritic distribution. Loss of Pea3 leads to ectopic expansion of these motorneuron dendrites into the central grey matter and also aberrant innervation by Ia afferents that would normally synapse only with Pea3 negative motor pools [6].

The way in which motorneuron dendrites attain their particular morphologies and territories so as to form myotopic maps has not been resolved. Much of what we know about dendrite development derives from work on sensory systems, and in general, the shapes of dendritic trees emerge as the product of intrinsic cell specification programmes and interactions with extrinsic cues and neural activity (reviewed in [9],[10]). How dendrites are positioned in particular layers or territories remains incompletely understood. In the mammalian retina, for example, layer-specific innervation of retinal ganglion cell dendrites relies on activity-dependent dendritic pruning and consolidation [11],[12], while in zebrafish most retinal ganglion cells put their dendrites directly into appropriate target laminae [13] and stratification of the inner plexiform layer occurs in the absence of neural activity [14]. Such “hard-wiring” is also evident in the *Drosophila* olfactory system, where the graded expression of Semaphorin-1a in projection neuron dendrites contributes to the formation of a sensory map in the antennal lobe [15],[16].

In this work, we have studied the mechanisms in a motor system that underlie the generation of different dendritic morphologies and the targeting of dendrites to distinct territories. We used the locomotor system of the *Drosophila* embryo, currently the only model system in which an explicit myotopic distribution of dendrites has been demonstrated [3]. First, we show that the internal muscle motorneurons fall into three morphological classes that have dendrites in distinct territories with respect to the ventral midline. These medio-lateral dendritic domains are arranged to form a myotopic representation of the muscle field. Second, we demonstrate that this myotopic map is generated by dendritic targeting and that it forms in the absence of excitatory neurotransmission and when presynaptic partner terminals have been displaced. Third, we have identified Robo and Frazzled signalling in the motorneurons as the key mechanism for dendritic targeting and map formation. Fourth, a detailed quantitative analysis of dendritic trees reveals that the programmes specifying dendritic growth and targeting are separable. Neurons generate a cell type–specific amount of dendritic length and number of branch points, while the combinatorial action of Robo and Frazzled implements the distribution of the available dendritic material in response to the midline derived cues Slit and Netrin, respectively. Just as we have demonstrated here for postsynaptic dendrites, global guidance has previously been shown to also position presynaptic sensory terminals in distinct neuropile regions [17],[18]. We therefore suggest that such global patterning cues organise connectivity in that they coordinate the delivery of pre- and postsynaptic partner terminals to common regions.

## Results

### Three Classes of Motorneurons with Distinct Dendritic Morphologies and Territories Form a Neural Map of the Body Wall Musculature

We set out to investigate how different dendritic morphologies and territories are generated in a motor system, using the neuromuscular system of the *Drosophila* embryo as a model. Its principal components are segmentally repeated arrays of body wall muscles (30 per abdominal half segment), each innervated by a specific motorneuron [19],[20]. The motorneuron dendrites are the substrate on which connections with presynaptic cholinergic interneurons form [21]–[25]. We labelled 180 cells (on average 11.25 for each identified motorneuron and a minimum of five) and charted the dendritic morphologies and territories of the motorneurons that innervate the internal muscles (see Table S1 and Figure S1), using retrograde labelling with the lipophilic tracer dyes “DiI” and “DiD.” We did so in the context of independent landmarks, a set of Fasciclin 2-positive axon bundles [26], at 18.5 h after egg laying (AEL), when the motor system first becomes robustly functional [21],[27] and the geometry of motorneuron dendritic trees has become sufficiently invariant to permit quantitative comparisons [25].

**Figure 1:**
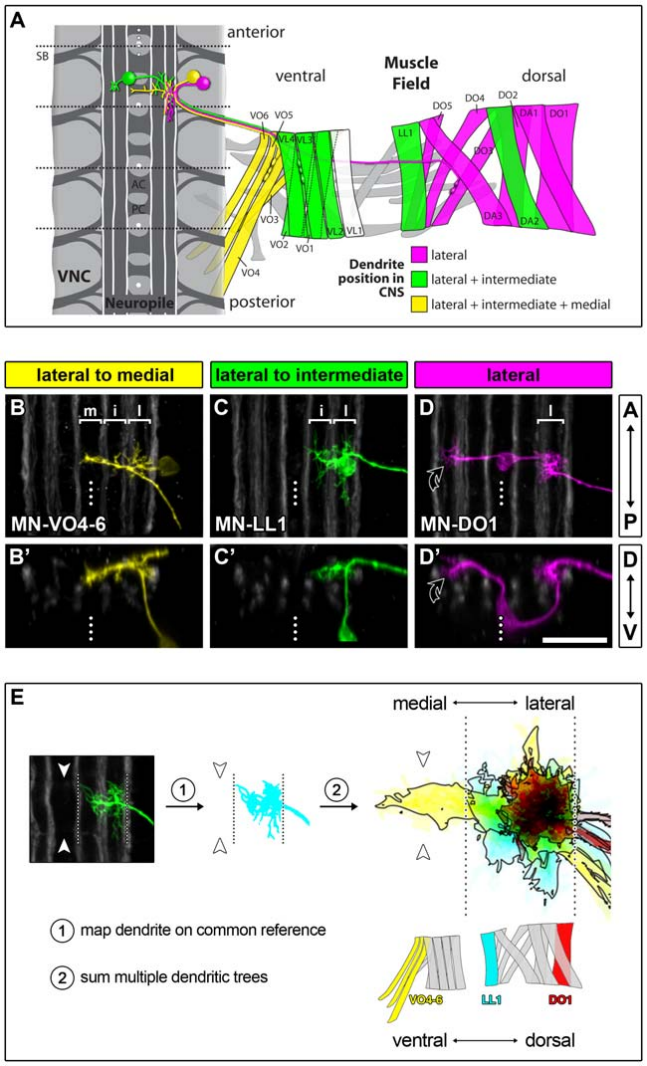
Central representation of the muscle field by motorneuron dendrites. The diagram shows the abdominal neuromuscular organisation of the *Drosophila* embryo at ∼18.5 h AEL (A). Muscles are colour-coded according to the lateral-to-medial dendritic extent of their specific motorneurons. Muscle VL1 is white because we have not been able to identify a specific type-Ib motorneuron for this muscle at embryonic stages. Motorneurons innervating the external muscle field (grey) were not considered in this study. *SB*, segment boundary; *AC*, anterior commissure; *PC*, posterior commissure. The white dotted line indicates the CNS midline. Muscle nomenclature is according to [82]: *DA*, dorsal acute; *DO*, dorsal oblique; *LL*, lateral longitudinal; *VL*, ventral longitudinal; *VO*, ventral oblique. The micrographs show projection-views ([B–D] z-projections, anterior up; [B′–D′] cross-sections, dorsal up) of dendritic arborisations of DiI/DiD-labelled, pseudo-coloured motorneurons (motorneurons are named according to the target muscles that they innervate) in the context of Fasciclin2-GFP-positive axon bundles (white) at 18.5 h AEL. Motorneurons with similar medio-lateral dendritic fields innervate muscles of related dorso-ventral position: (B) lateral (l), intermediate (i), and medial/midline (m) targeting dendrites, yellow; (C) lateral and intermediate dendrites, green; (D) laterally confined dendrites, magenta. MN-DO1 and three other internal motorneurons each have an additional contralateral dendritic subtree ([D, D′] black curved arrow) that generally reflects the distribution of the main ipsilateral arbor. Scale bar: 20 µm. (E) “Cumulative plots” of motorneuron dendritic trees at 18.5 h AEL were generated using the lateral and medial Fasciclin2-positive axon tracts as reference lines (thin dotted lines) to map multiple dendritic trees onto a common reference grid. Dendrite plots for three representative motorneurons (MN-DO1, red, *n* = 4; MN-LL1, cyan, *n* = 19; MN-VO4–6, yellow, *n* = 5) are shown to illustrate the “myotopic” organisation principle. Arrowheads indicate the midline.

We find that there are three classes of motorneurons based on dendritic arbor morphology and territory with respect to the ventral midline: i) motorneurons with dendrites in the lateral neuropile (between the lateral and intermediate Fasciclin 2 tracts), ii) in the lateral and intermediate neuropile (between the intermediate and medial Fasciclin 2 tracts), and iii) in the lateral, intermediate plus medial neuropile (posterior commissure) (Figure 1).

Moreover, the medio-lateral positions of motorneuron dendrites correlate with the dorsal to ventral locations of their target muscles in the periphery. Motorneurons with dorsal targets (DA1, DA3, DO1–5) have their dendrites in the lateral neuropile, while those innervating ventral and lateral muscles (LL1, VL2–4, VO1–2) also have dendrites in the intermediate neuropile. Coverage of the medial neuropile is particular to motorneurons innervating the most ventral group of muscles (VO3–6). These dendritic domains are arranged in the medio-lateral axis of the neuropile in such a way that they form a neural, myotopic representation of the distribution of body wall muscles in the periphery (Figure 1). Only a single motorneuron deviates from this clear-cut correlation between dendritic medio-lateral position and target muscle location: MN-DA2 has dendrites not only in the lateral neuropile, like other motorneurons with dorsal targets, but also in the intermediate neuropile (see also Figure S2 for all internal motorneurons).

### Motorneurons Target their Dendrites to Specific Medio-Lateral Territories

Having identified an experimental framework with clear, reproducible distinctions between dendritic morphologies and territories, we sought to identify the mechanisms that underlie the generation of these differences. Neurons can acquire characteristic dendritic geometries by different strategies. Dendrites might grow out radially, in a random fashion, so that the dendritic territory emerges as some branches are maintained while other, inappropriately targeted segments are pruned back. This process of radial exploration is thought to involve selective stabilisation of branches by synaptic contact and/or transmission [10]–[12]. Alternatively, dendritic growth may be biased towards a particular direction or area in response to guidance cues [16],[28],[29]. To distinguish between these two alternatives, we established a developmental time line, comparing dendritic territories at different developmental stages: i) 15 h AEL, 1 h before synaptic connections first become functional; ii) 18.5 h AEL, when the motor system is first robustly operational; and iii) 21 h AEL (hatching), when the system is mature [21].

**Figure 2:**
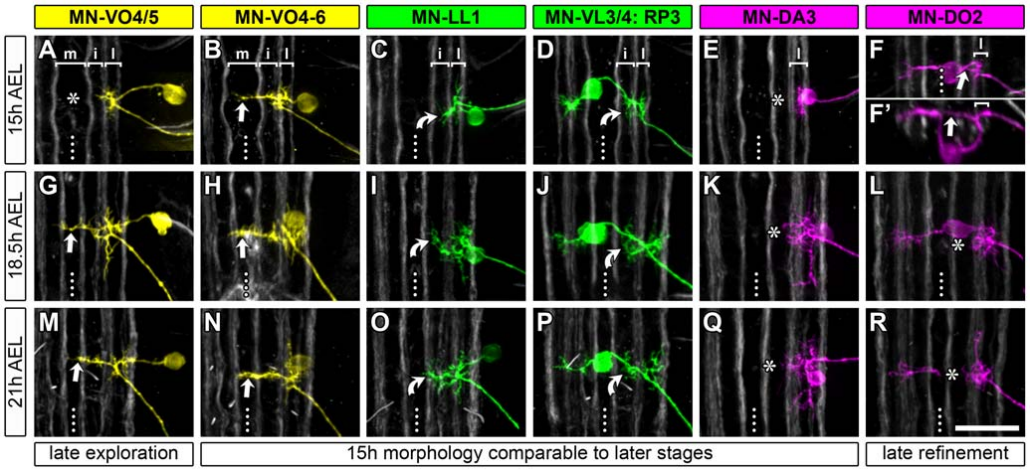
Development of medio-lateral dendritic patterns between 15 h and 21 h AEL. Single DiI/DiD-labelled dendritic trees of six representative motorneurons are shown at three different time-points during embryonic development (15 h, 18.5 h, and 21 h AEL). (B–E, H–K, N–Q) In most cases, dendritic territories characteristic for developmental stages when the motor system is functional (18.5 h AEL) have already become apparent by 15 h AEL. Curved arrows indicate dendrites in the intermediate territory and straight arrows dendrites in the medial neuropile. (A, G, M) MN-VO4/5 has “late exploring” dendrites that innervate the midline neuropile (straight arrow) between 15 h and 18.5 h AEL (“late exploration”; compare [A and G]; asterisk indicates absence of midline branches at 15 h AEL). (F, L, R) MN-DO2 dendrites on the other hand appear to undergo “late refinement” between 15 h and 18.5 h AEL. Branches are seen reaching into the intermediate/medial neuropile at 15 h AEL ([F, F′] straight arrow) but by 18.5 h AEL all MN-DO2 dendrites are confined to the lateral neuropile (“late refinement”; asterisks in [L and R] indicate intermediate/medial neuropile devoid of MN-DO2 dendrites at 18.5 h and 21 h AEL). Because the MN-DO2 cell body can obscure dendritic trees in projection views a cross section view is also shown in (F′) and optical slices that would have shown the ventrally located cell body have been omitted from the projection shown in (R) so that the dendrites can be seen clearly. In general, motorneuron dendritic trees have markedly increased in size from 15 h to 18.5 h AEL. Between 18.5 h and 21 h AEL (hatching) further adjustments in dendritic extent, morphology, and position occur but are more subtle. For instance, at 18.5 h AEL 7/9 labelled MN-VO4/5 show posteriorly projecting dendritic branches to varying degrees (see [G] for an example with a pronounced posterior dendritic projection) that are not seen at 21 h AEL (*n* = 6). In all micrographs anterior is up except for (F′) where dorsal is up. Dotted lines indicate CNS midlines. Scale bar: 20 µm.

At 15 h AEL dendritic trees are more variable though less extensive than at 18.5 h AEL. In the majority of cases (12/16 cell types) motorneuron dendritic arbors have already generated the morphology and have invaded the neuropile territories that are characteristic of their more mature 18.5 h counterparts (Figure 2). For instance, MN-DA1 (aCC), MNs-DO3–5, and MN-DA3 have predominantly laterally located dendrites at 15 h (87.5% have entirely laterally located dendrites; *n* = 24 labelled cells), as at 18.5 h AEL (100% with entirely laterally positioned dendrites for MN-DA1, *n* = 9 fills; 77.8% for MNs-DO3–5, *n* = 18 fills; 20% for MN-DA3, *n* = 24 fills, Figure 2). Dendritic innervation of lateral and intermediate (MN-DA2, MN-LL1, MN-VO1 [RP4], MN-VO2 [RP1], MN-VL2, MN-VL3/4 [RP3]) or lateral to medial (MN-VO4–6) neuropile territories is already apparent at 15 h AEL (*n* = 38 fills) and consistently still present at 18.5 h AEL (*n* = 95 fills; Figure 2). Changes in dendritic territories between 15 h and 18.5 h AEL were manifest for only 4/16 of the motorneurons. Disappearance of dendritic branches transiently located in the intermediate neuropile was evident for MN-DO1 and MN-DO2: at 15 h AEL 64% of the two cells (*n* = 11) had dendritic branches in the intermediate neuropile, while at 18.5 h AEL all MN-DO2 and all but one of the MN-DO1 dendrites were confined laterally (*n* = 15). Late exploration of the midline neuropile was seen for two other motorneurons: at 18.5 h AEL MN-VO3 and MN-VO4/5 have characteristic midline-targeting dendrites (*n* = 19), which are never present earlier, at 15 h AEL (*n* = 8; Figure 2).

We next asked what dendritic changes might occur between 18.5 h AEL and hatching at 21 h AEL. Based on a subset of nine representative motorneurons (MN-DO1 [*n* = 5], MN-DO2 [*n* = 1], MN-DO3 [*n* = 2], MN-DA3 [*n* = 8], MN-LL1 [*n* = 13], MN-VL2 [*n* = 1], RP3 [*n* = 2], MN-VO4/5 [*n* = 11], MN-VO4–6 [*n* = 5]) we see no substantial change in the overall morphology of dendritic trees or their medio-lateral territories between 18.5 h and at 21 h AEL (Figure 2).

In summary, we find that already at 15 h AEL, before the onset of synaptic input, 75% (12/16) of the internal muscle motorneuron types have their dendrites located in and confined to the territories that are characteristic of the functional (18.5 h AEL) and mature system (21 h AEL, hatching). This suggests, at least for the majority of motorneurons, that synaptic activity is probably not required for targeting dendrites to particular domains.

### The Dendritic Myotopic Map Forms in the Absence of Excitatory Synaptic Transmission

To test directly whether synaptic transmission is indeed dispensable for the formation of the dendritic myotopic map, we visualised motorneurons in *cha^l13^* mutant embryos [30] at 18.5 h AEL. These embryos fail to synthesize acetylcholine, which is the main, and at this stage probably exclusive, excitatory neurotransmitter for motorneurons [21],[22]. Cholinergic synaptic input onto motorneurons normally commences at 16 h AEL [21] and negatively regulates dendritic growth [25].

**Figure 3:**
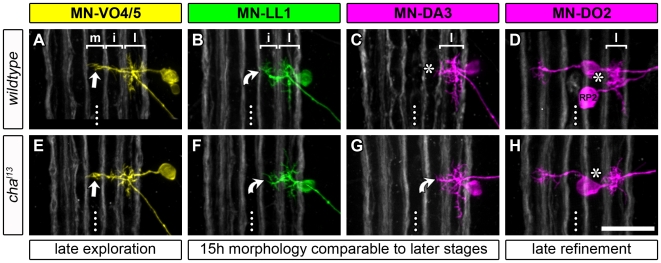
Medio-lateral dendritic targeting in the absence of cholinergic synaptic transmission. Single DiI/DiD-labelled dendritic trees of four representative motorneurons (as described in Figure 2) are shown at 18.5 h AEL in wild-type and *cha* mutant embryos. (A, E) By 18.5 h AEL, both in the control and mutant condition, MN-VO4/5 has established its characteristic medial dendritic subtree (straight arrow), respectively (*n* = 9). (D, H) MN-DO2 (*n* = 3) and MN-DO1 (*n* = 10; not shown) dendrites are strictly confined to the lateral neuropile in *cha* mutant embryos as in the wild-type (note that in [D] the “common exciter” RP2 was also labelled). (B, C) MN-LL1 and MN-DA3 can normally be clearly distinguished: MN-LL1 has manifest branches innervating the intermediate neuropile (curved arrow), which are not formed by MN-DA3 (asterisk). (F, G) In 18.5 h *cha* mutants, some MN-LL1 and MN-DA3 cells form dendrites that are less distinct than in the wild-type and examples of such cases are shown here: in 3/10 cases MN-LL1 dendrites in the intermediate territory were less extensive than in the wild-type (curved arrow in [F], compare with [B and G]); in 4/11 cases MN-DA3 dendrites extended slightly more medially than in controls (curved arrow in [G], compare with [C and F]). Asterisks in (C, D, and H) indicate intermediate neuropile devoid of dendritic branches. Dotted lines indicate CNS midlines. Scale bar: 20 µm.

We analysed internal muscle motorneurons representative of the three modes of establishing dendritic territories: i) “late refining” MN-DO1 (*n* = 10) and MN-DO2 (*n* = 3) transiently put some dendritic branches “inappropriately” into the intermediate neuropile before confining these to the lateral neuropile by 18.5 h AEL; ii) “late exploring” MN-VO4/5 (*n* = 9) has dendrites that invade the midline neuropile relatively late, during the 15–18.5 h interval; iii) MN-DA3 (*n* = 11), MN-LL1 (*n* = 10), and MN-VO4–6 (*n* = 6) have already attained their characteristic dendritic territories by 15 h AEL. In the absence of acetylcholine we detected changes in the dendritic arbors in a fraction of MN-DA3 and MN-LL1 cells, as compared to wild-type. 4/11 MN-DA3 had larger and 3/10 MN-LL1 had smaller than normal dendritic arborisations in the intermediate neuropile. However, all other motorneurons studied in 18.5 h *cha^l13^* mutant embryos had dendritic morphologies and innervated territories that were comparable to wild-type (Figure 3). This shows that excitatory synaptic input is not essential for the development of normal overall motorneuron dendrite morphology or the formation of the myotopic map.

### Presynaptic Terminals Do Not Provide Patterning Information for the Dendritic Map

**Figure 4:**
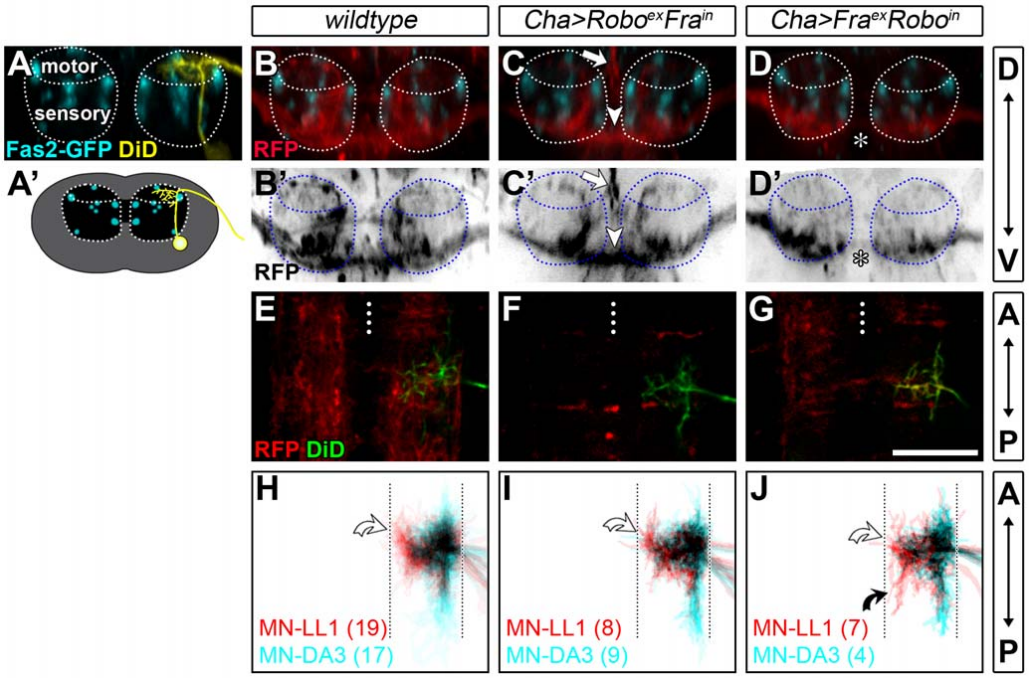
Normal positioning and contact with presynaptic cholinergic terminals is not required for dendritic medio-lateral targeting. (A) Digital cross section from a confocal stack and a schematic cross section (A′) of a motorneuron labelled with DiD (yellow) in the ventral nerve cord relative to the Fasciclin2-GFP-positive tracts (cyan) at 18.5 h AEL. The motorneuron dendrites arborise in the dorsal (motor) neuropile whereas sensory axons primarily terminate in the ventral neuropile. (B–D′) show corresponding cross sections of nerve cords in which cholinergic processes are visualised by expression of membrane targeted *UAS-myr-mRFP* using *Cha-GAL4* (red) in the context of Fasciclin2-GFP-positive tracts (cyan). In addition, chimeric Robo-Fra receptors were expressed to displace the cholinergic terminals (C–D′). (C, C′) Expression of the Robo^ex^Fra^in^ receptor leads to a thickening of the commissural cholinergic tracts ventrally (arrowheads) and an accumulation of cholinergic terminals dorsally at the midline (straight arrows). (D, D′) Conversely, expression of the repulsive receptor Fra^ex^Robo^in^ induces a severe depletion of cholinergic fibers from the commissures (asterisks). (E–G) Single confocal slices at a position in the dorsal neuropile where motorneuron dendrites (labelled with DiD, green) form show a marked decrease in cholinergic innervation (red) when the chimeric receptors are expressed (compare [E] with [F and G]; note that in [G] the dendritic tree was extremely brightly labelled resulting in a fraction of the DiD signal being picked up in the red RFP channel so that the overlay of both channels appears yellow in places). (H–J) Cumulative plots of MN-LL1 and MN-DA3 dendritic trees in controls (H) and under experimental conditions (I, J) show that their medio-lateral distinctions are clearly apparent, as in the wild-type ([H], n-numbers given in parentheses). Unlike MN-DA3, MN-LL1 reproducibly targets the intermediate neuropile (white curved arrows in [H–J]). However, in 4/7 cases MN-LL1 formed abnormal posteriorly projecting branches in the intermediate neuropile in *Cha-GAL4; UAS-Fra^ex^Robo^in^* embryos (black curved arrow in [J]). D, dorsal; V, ventral; A, anterior; P, posterior. Dotted lines indicate CNS midlines. Scale bar: 20 µm.

The dendritic trees of motorneurons form within an existing scaffold of interneuron axons and we previously found evidence for dendritic growth being regulated by contact with presynaptic partner terminals [25]. We therefore asked if the presynaptic partner terminals might provide patterning information for the dendritic myotopic map. To this end, we displaced the presynaptic partner axons, contained in the set of cholinergic interneurons [22], by expression of two potent chimeric axon guidance receptors: *UAS-Fra^extracellular^-Robo^intracellular^-myc* (*UAS-Fra^ex^Ro^in^*), shown to shift axons away from the midline, and *UAS-Robo^extracellular^-Fra^intracellular^-myc* (*UAS-Ro^ex^Fra^in^*), which can mediate the opposite effect [31]. As expected, expression of *UAS-Fra^ex^Ro^in^* with *Cha-GAL4* leads to a severe depletion or absence of cholinergic axons in the commissures and expression of *UAS-Ro^ex^Fra^in^* to a thickening of commissural cholinergic tracts (Figure 4B–4D′; see also [25]). In addition, we find that expression of either chimeric construct efficiently displaces cholinergic axon terminals out of the dorsal motor neuropile. Under these conditions contact of motorneuron dendrites with cholinergic interneuron terminals is severely reduced and potentially absent at 18.5 h AEL, unlike in the wild-type (Figure 4E–4G); yet the overall organization of the neuropile, including the distribution of Fasciclin 2-positive tracts, is not obviously affected (see also [25]). We find that these manipulations do not obviously affect midline targeting of dendrites by motorneurons that innervate ventral oblique muscles (*n* = 4, unpublished data). Dendritic intermediate (MN-LL1; white curved arrows) and lateral (MN-DA3) territories also remain distinct, though appear more variable as compared to controls (Figure 4H–4J).

We conclude that the presynaptic partner axons do not provide positional information necessary for the myotopic map of motorneuron dendrites. However, the increased variability observed under these experimental conditions suggests interactions with presynaptic partners may influence finer aspects of dendritic arbors as previously shown [25].

### Midline Guidance Cues Direct Dendritic Targeting to the Midline Territory

Next we asked whether the different distributions of motorneuron dendrites with respect to the ventral midline resulted from different responses to midline derived guidance cues. Obvious candidates are Slit and Netrins and their respective receptors, Robo and Frazzled. These have been shown to regulate the position of outgrowing axons by attraction (Netrin-Frazzled) and repulsion (Netrin-Unc5, Slit-Robo; reviewed by [32]) and to gate midline crossing of dendrites in the *Drosophila* embryo, larva, and adult [28],[33],[34].

**Figure 5:**
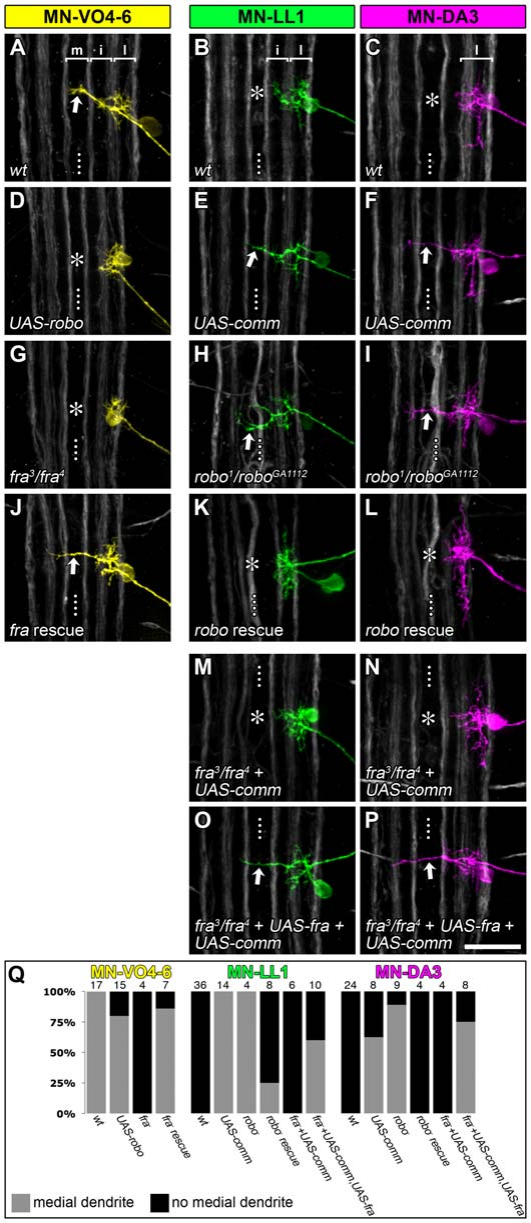
Frazzled and Robo are required cell-autonomously and gate dendritic targeting to the midline. Single DiI/DiD-labelled dendritic trees of MN-DA3, MN-LL1, and MN-VO4–6 at 18.5 h AEL in controls and when levels of Frazzled and/or Robo have been altered. The midline targeting dendrite of MN-VO4–6 ([A] straight arrow) fails to form when *UAS-robo* is expressed (using *CQ-GAL4* [D]) or in a *fra*-mutant background (G). The *fra*-mutant phenotype can be rescued by reinstating *UAS-fra* with *CQ-GAL4* ([J] straight arrow). Conversely, in MN-DA3 and MN-LL1 (B and C for wild-type) inactivation of Robo (*CQ-GAL4* driving *UAS-comm* or *robo^1^/robo^GA1112^*) produces a usually single aberrant midline targeting dendritic branch per cell ([E, F, H, I] straight arrows). The *robo*-mutant phenotype can be rescued by driving *UAS-robo* with *CQ-GAL4* (K, L). The *UAS-comm* phenotype is suppressed in a *fra*-mutant background (asterisks in [M, N] indicate the absence of midline targeting branches; compare with [E, F]) and recovered by co-expressing additionally *UAS-fra* in MN-DA3 and MN-LL1 ([O, P] straight arrows). (Q) illustrates the distribution of dendritic phenotypes for each motorneuron and genotype with indicated n-numbers above each bar. Dotted line: CNS midline. Scale bar: 20 µm.

We focused on three motorneurons, each representing one of the three principal classes of dendritic morphology and medio-lateral territories: i) MN-VO4–6 has dendrites in the lateral, intermediate, and midline neuropile (Figure 5A); ii) MN-LL1 has only lateral and intermediate dendrites (Figure 5B); and iii) MN-DA3 dendrites are located in the lateral neuropile (Figure 5C). These neurons meet two additional criteria: first, their axons do not traverse the dorsal neuropile, so that one can clearly differentiate between dendrite morphogenesis and axonal (collateral) outgrowth; secondly, they can be manipulated genetically with great specificity using the *CQ-GAL4* expression line. Use of this expression line does not obviously interfere with motor axon pathfinding or target recognition, as assayed by differential labelling of multiple motorneurons in a segment and the presence and distribution of neuromuscular junctions in the periphery (unpublished data).

In the first instance, we investigated dendritic targeting to the midline territory. We tested if exclusion of MN-LL1 and MN-DA3 dendrites from the midline neuropile was implemented by the presence of Robo in these cells. To this end we down-regulated Robo in MN-LL1 and MN-DA3 through targeted expression of *UAS-comm* [35],[36] and found this to induce ectopic dendritic innervation of the midline neuropile (Figure 5E, 5F, and 5Q; 100% penetrance for MN-LL1, *n* = 14; 62.5% for MN-DA3, *n* = 8). Loss of Robo in embryos entirely mutant for *robo* (*robo^1^/robo^GA1112^*) also generates a comparable ectopic midline innervation phenotype (Figure 5H, 5I, and 5Q; 100% penetrance for MN-LL1, *n* = 4; 89% for MN-DA3, *n* = 9), and this can be rescued by reinstating *robo* selectively in MN-LL1 and MN-DA3 using *CQ-GAL4* (Figure 5K, 5L, and 5Q) (rescue efficiency: 75% for MN-LL1, *n* = 8; 100% for MN-DA3, *n* = 4).

Conversely, we find that expression of the Slit receptor Robo in MN-VO4–6 can abolish dendritic targeting to the midline (20% penetrance, *n* = 15) (Figure 5D and 5Q), a phenotype that we have never observed in the wild-type at 18.5 h AEL (*n* = 17). We suspect that the low penetrance in this particular case is due to low GAL4 activity in MN-VO4–6 at the time when its dendrites first explore the midline, and perhaps endogenous *comm* expression, which would normally permit dendritic growth to the midline and antagonize the effects of ectopically expressed Robo. In embryos mutant for the Netrin receptor Frazzled (*fra^3^/fra^4^*), MN-VO4–6 shows a comparable phenotype albeit at high penetrance (as does MN-VO4/5; 100% penetrance, *n* = 11 for these two cells), and this can be rescued by reinstating Frazzled in MN-VO4–6 (86% penetrance, *n* = 7) (Figure 5G, 5J, and 5Q).

These results show that cell-autonomous expression of guidance cue receptors is necessary (for Frazzled) and sufficient (for Robo) to gate the growth of motorneuron dendrites to the midline neuropile. The data further suggest that dendrite growth to the midline requires not only a lack (or low levels) of Robo-mediated repulsion but also attraction mediated by Frazzled. We confirmed an absolute requirement for Frazzled. MN-DA3 and MN-LL1 dendrites fail to innervate the midline neuropile upon down-regulation of Robo when Frazzled is also absent (100% penetrance, *n* = 10; Figure 5M, 5N, and 5Q) but ectopically target the midline when Frazzled expression is selectively reinstated in these cells (66% penetrance, *n* = 18; Figure 5O, 5P, and 5Q).

### Intermediate and Lateral Dendritic Territories Are Specified by the Balance of Robo and Frazzled Signalling

**Figure 6:**
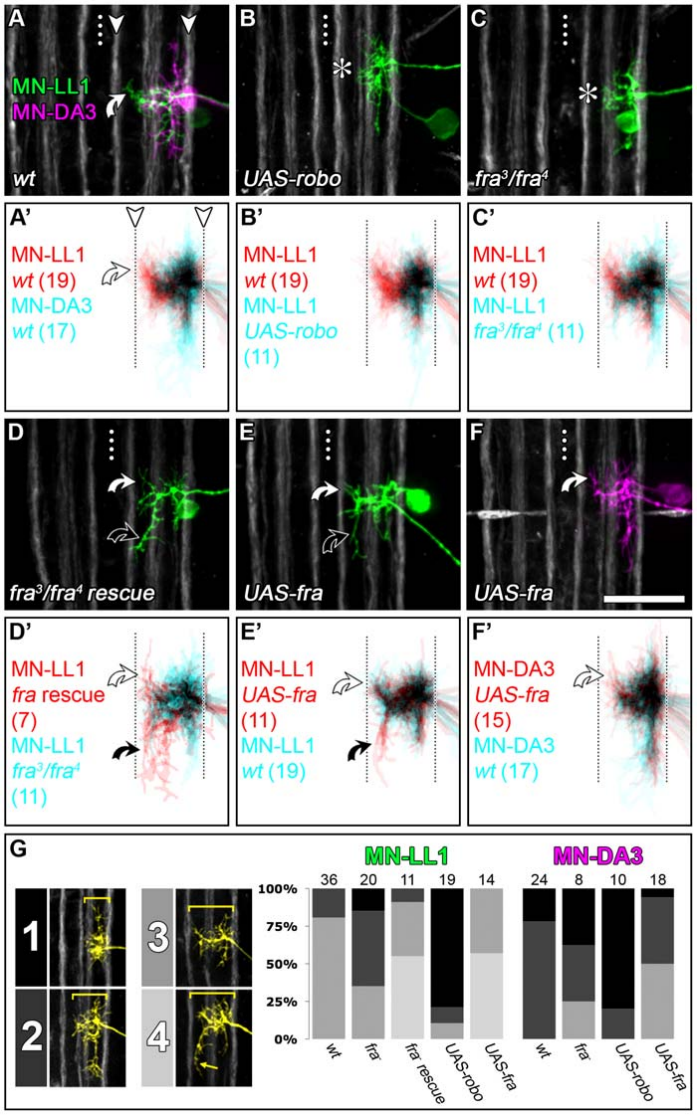
Robo and Frazzled mediate dendritic targeting in the intermediate and lateral neuropile. (A–F) DiI/DiD-labellings of single MN-LL1 and MN-DA3 at 18.5 h AEL in *fra-* or *robo-*manipulated genetic backgrounds. (A′–F′) Cumulative plots generated from z-projections of various cells that were mapped onto a common reference grid using Fasciclin2-GFP-positive axon bundles as landmarks and shown in two channels, each representing one of two experimental conditions (n-numbers of cells in each plot are given in parentheses). Saturated colours indicate highly reproducible dendritic coverage at the respective relative position. In the wild-type, MN-LL1 can be distinguished from MN-DA3 by the presence of a dendritic subtree located in the intermediate neuropile (white curved arrow in [A, A′]). (B–C′) In *fra* mutants (C, C′) and when *UAS-robo* is expressed (B, B′; using *CQ-GAL4*) the intermediate dendrites of MN-LL1 fail to form (asterisks in [B and C]). (D, D′) Cell-specific expression of *UAS-fra* in the *fra*-mutant background rescues the intermediate MN-LL1 dendrites (white curved arrows) and frequently generates an ectopic posteriorly projecting intermediate branch (black curved arrows). (E, E′) Similarly, expression of *UAS-fra* in a wild-type background produces ectopic posteriorly projecting intermediate dendrites in MN-LL1 (black curved arrows). (F, F′) *UAS-fra* expression in MN-DA3 results in a subtle ectopic innervation of the intermediate neuropile (white curved arrows). (G) Illustration of and penetrance of four distinct medio-lateral dendritic morphologies for each motorneuron and genotype with indicated n-numbers above each bar. Dotted line: CNS midline. Scale bar 20 µm.

We then examined how the distinction between the intermediate and lateral dendritic territories is specified at a distance (approx. 5–10 µm) from the ventral midline. For instance, the distinguishing feature between MN-DA3 and MN-LL1 is that MN-LL1 has an additional dendritic sub-arbor in the intermediate neuropile (Figure 6A and 6A′; white curved arrow). To test whether Robo-Slit signalling in dendrites might also define this distinction between intermediate and lateral neuropile territories, we expressed Robo selectively in MN-LL1 and MN-DA3. We find that increasing the levels of Robo in MN-LL1 reliably converts its dendritic tree to a MN-DA3-like morphology in 15/19 cases (Figure 6B, 6B′, and 6G). For MN-DA3, this manipulation enhances the characteristic lateral confinement of its dendrites in 8/10 cases (Figure 6G).

We next tested the role of Frazzled-mediated attraction in generating the distinction between intermediate (MN-LL1) and lateral (MN-DA3) dendritic territories. To this end we removed, reinstated, and overexpressed *frazzled* in MN-LL1 and MN-DA3. In *fra^3^/fra^4^* mutant embryos, 63% of MN-LL1 dendritic arbors lack the normally pronounced intermediate dendritic arborisation (*n* = 20; Figure 6C, 6C′, and 6G), while targeting of MN-DA3 dendrites is not significantly affected (*n* = 8; Figure 6G). Reinstating *frazzled* selectively in MN-LL1 in *fra^3^/fra^4^* mutant embryos efficiently rescues dendritic targeting to the intermediate neuropile (*n* = 11). Moreover, this manipulation leads to a greater proportion of dendritic branches innervating the intermediate neuropile (Figure 6D and 6D′; black curved arrows), as does overexpression of Frazzled in an otherwise wild-type background (57% of cases, *n* = 14; Figure 6E and 6E′; black curved arrows). For MN-DA3 expression of *UAS-fra* leads to ectopic innervation of the intermediate neuropile in 50% of cases, converting the dendritic arbor to a MN-LL1-like morphology (*n* = 18; Figure 6F and 6F′; white curved arrows). Frazzled overexpression in MN-LL1 or MN-DA3 never led to ectopic midline targeting of dendrites (*n* = 44).

Last, we tested the requirement for Robo and Frazzled signalling in motorneurons for setting up dendritic medio-lateral territories at an earlier stage, when dendritic domains first become recognizably distinct [3]. At 15 h AEL we find the same requirement as at 18.5 h AEL for the combinatorial action of Robo and Frazzled in motorneurons in the three dendritic territories with respect to the ventral midline (Figure S3).

These results suggest that Robo and Frazzled are the key factors, whose relative levels in motorneurons determine the distinction between lateral and intermediate dendritic territories. Lateral confinement of dendrites (e.g., MN-DA3) can be achieved either by high levels of Robo and/or low levels of Frazzled expression. Targeting to the intermediate (but not midline) neuropile (e.g., MN-LL1) requires relatively high levels of Frazzled and low Robo activity.

### Frazzled Mediates Netrin Attraction in Dendrites

**Figure 7:**
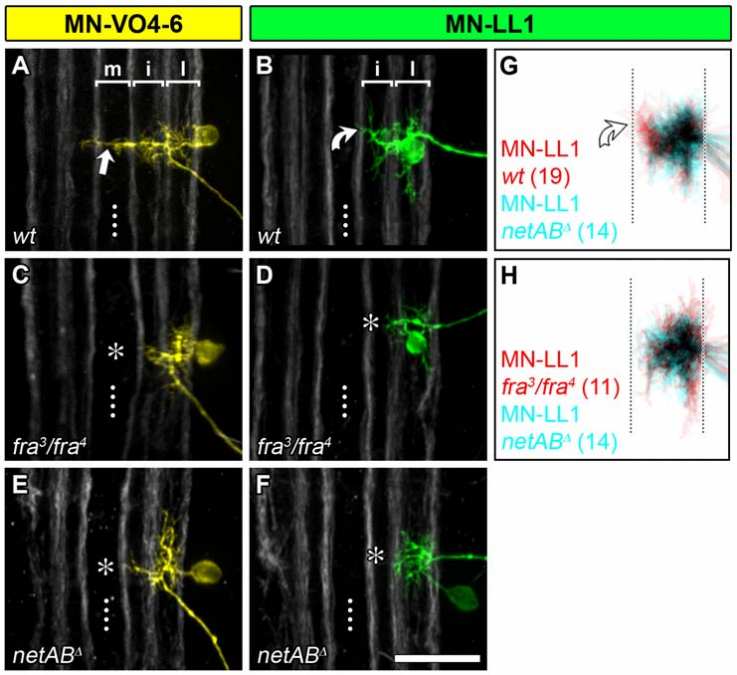
Dendritic *frazzled* and *netrin*-phenotypes are comparable. (A–F) DiI/DiD-labellings of single MN-VO4–6 and MN-LL1 at 18.5 h AEL in control, *fra*-, and *netAB^Δ^*-mutant embryos, as indicated. (G, H) cumulative plots generated from z-projections of these cells (n-numbers are given in parentheses). The *fra* and *netAB^Δ^* dendritic phenotypes are indistinguishable: MN-VO4–6 normally forms characteristic midline-targeting dendrites (straight white arrow in [A]) that are missing in *fra* and *netAB^Δ^* mutants (asterisks in [C and E] indicate missing midline targeting dendrites). MN-LL1 dendrites normally innervate the intermediate neuropile (curved arrow in [B]), but this innervation is either absent or clearly reduced in ∼65% of cases in the mutants (asterisks in [D and F]). The congruent overlap of cumulative MN-LL1 plots from *fra* and *netAB^Δ^* mutants shows that the dendritic phenotypes are virtually identical (H). Asterisks in (C–F) indicate intermediate and medial neuropile devoid of dendritic branches. Dotted line: CNS midline. Scale bar 20 µm.

Our data show that Frazzled is absolutely required for dendritic growth to the midline. However, other Netrin receptors, such as Unc5 [37] and Dscam [38],[39], have been identified, as well as Netrin independent midline guidance systems [40],[41]. To determine whether Frazzled is the main Netrin receptor for dendritic targeting and Netrin the sole attractant, we asked if loss of Frazzled produced the same dendritic phenotype as the loss of Netrin. This is indeed the case. In *netAB^Δ^* mutants [40] MN-VO4–6 dendrites fail to target the midline neuropile (100% penetrance, *n* = 6) precisely as in *fra^3^/fra^4^* mutants (100% penetrance, *n* = 4; MN-VO4/5 has the same mutant phenotype, *n* = 7) (Figure 7A, 7C, and 7E). Similarly, MN-LL1 has a clearly reduced innervation of the intermediate neuropile in 63%–64% of cases in both *netAB^Δ^* and *fra^3^/fra^4^* mutant embryos (*n* = 14 for *netAB^Δ^*; *n* = 20 for *fra^3^/fra^4^*) (Figure 7B, 7D, and 7F–7H).

These observations suggest that in the embryonic nerve cord attraction of motorneuron dendrites to the ventral midline is mediated primarily, if not exclusively, by a Frazzled-containing receptor complex in response to Netrin. At the same time, we cannot entirely rule out that other ligand/receptor pairs might also contribute, though in more subtle ways, to positioning dendrites to the intermediate or midline neuropile.

### Dendritic Targeting Is Separable from the Cell-specific Programme of Dendritic Growth and Branching

**Figure 8:**
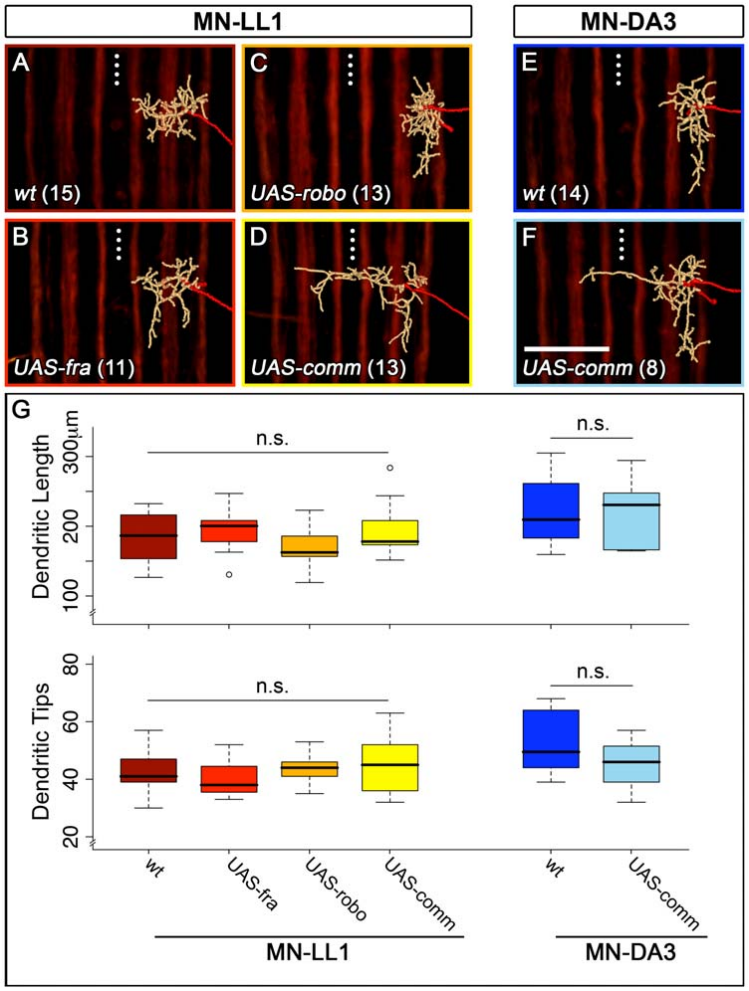
Dendritic targeting phenotypes are not associated with overall changes in dendritic length and tip numbers. Images of representative digitally reconstructed dendritic trees (beige; axons coloured red) of MN-LL1 (A–D) and MN-DA3 (E, F) in different genetic backgrounds (18.5 h AEL; transgenes were expressed using *CQ-GAL4*; n-numbers are given in parentheses). (G) Box plots illustrating the distribution of total dendritic tree length and dendritic tip number. The median is indicated by a thick black horizontal bar, and the 25^th^ and 75^th^ percentiles are the bottom and top line of each box, respectively. Whiskers show the extremes of each dataset and in two cases outliers are indicated by circles. Although the medio-lateral positions of the dendritic trees vary dramatically depending on the genotype, their overall lengths and tip numbers do not show significant differences (G). Student's *t* test and Wilcoxon test were used for statistical analysis as appropriate. Anterior is up. Dotted line: CNS midline. Scale bar 20 µm.

**Figure 9:**
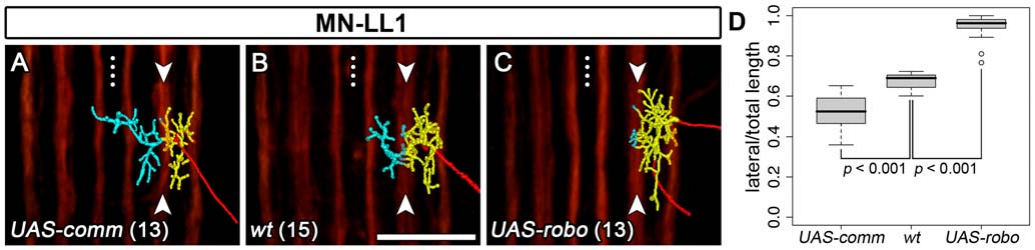
Genetic manipulations of Robo in MN-LL1 lead to a redistribution of dendritic branches. Images of representative digitally reconstructed dendritic trees of MN-LL1 under different experimental conditions ([A–C] 18.5 h AEL; transgenes were expressed using *CQ-GAL4*; n-numbers are given in parentheses). Each dendritic tree was subdivided into a lateral (yellow) and an intermediate/medial part (cyan) bisected by the intermediate Fasciclin2-GFP-positive tracts ([A–C] arrowheads). (D) Lengths of laterally located dendrites and total tree lengths were quantified individually and the ratios of lateral/total tree length are shown using box plots. The median is indicated by a thick black horizontal bar, and the 25^th^ and 75^th^ percentiles are the bottom and top line of each box, respectively. Whiskers show the extremes of each dataset and in two cases outliers are indicated by circles. Down-regulation of Robo by expression of *UAS-comm* in MN-LL1 induces ectopic dendritic innervation of the medial neuropile ([A] cyan) and concomitantly leads to a decrease in the extent of the arbor located laterally ([A] yellow). Conversely, expression of *UAS-robo* elicits the opposite effect, a reduction of the arbor that innervates the intermediate territory ([C] cyan) and an increase in the extent of the tree in the lateral neuropile ([C] yellow). Student's *t* test was used for statistical analysis. Anterior is up. Dotted line: CNS midline. Scale bar 20 µm.

Previous studies have shown that Frazzled/DCC-Netrin signalling can promote axonal growth [42],[43]. Others have implicated Robo-Slit signalling in regulating axonal and dendritic branching, the extension of axons, and the formation of dendrites [33],[44]–[47]. We therefore wanted to know how Frazzled and Robo signalling affects the growth and branching of motorneuron dendrites as it regulates their distribution in the neuropile. To this end, we quantified [48],[49] overall dendritic lengths and number of tips of MN-DA3 and MN-LL1 arbors under different experimental conditions (Figure 8). We find that the wild-type MN-DA3 and MN-LL1 reproducibly generate dendritic arbors with characteristically different total lengths (MN-DA3: 221.7 µm±47.7 *versus* MN-LL1: 183.1 µm±35.7; *t* test: *p* = 0.02) and tip numbers (MN-DA3: 53.0±10.0 *versus* MN-LL1: 42.8±7.6; *t* test: *p* = 0.005). Cell-specific loss- and gain-of-function of Robo (*UAS-comm* and *UAS-robo*) as well as overexpression of Frazzled reproducibly generates clear dendritic targeting phenotypes. However, these manipulations do not lead to statistically significant changes in overall dendritic length or tip number (Figure 8G). This indicates that Robo and Frazzled regulate the positioning of dendritic trees without noticeably affecting overall growth and branching. To test this hypothesis we focused on MN-LL1 and quantified the lengths of dendritic arbors located in the lateral neuropile as a percentage of total arbor length. Indeed, we find that changing the levels of Robo changes the proportion of the MN-LL1 dendritic tree that is put into the lateral neuropile. In the wild-type (*n* = 15), the average proportion of the dendritic tree in the lateral domain is 67.0%±4.3% (122.4 µm±24.2) of total tree length (183.1 µm±35.7) (Figure 9B and 9D). Down-regulation of Robo by *UAS-comm* expression (*n* = 13) induces ectopic dendritic growth towards the midline with a concomitant reduction of the lateral arbor to 52.5%±9.5% (101.6 µm±29.0; total length: 193.3 µm±39.3) (Figure 9A and 9D). Conversely, expression of *UAS-robo* (*n* = 13) reduces innervation of the intermediate neuropile and leads to a greater proportion of the arbor to be located in the lateral territory, namely 93.8%±7.3% (156.2 µm±32.1) of the total length (166.3 µm±29.8) (Figure 9C and 9D).

These quantifications suggest that, at least in the embryo, central neurons move towards generating a cell type–specific amount of dendritic length with a particular frequency of branching events. Guidance cue receptors act to distribute “available” dendrites, probably by locally modulating the rate of growth and/or stability of individual branches. At the same time, our data point to the existence of mechanisms that integrate such local changes across the entire arbor, since we do not observe statistically significant changes in overall tree length under different conditions.

### Excitatory Synaptic Contacts Form on Motorneuron Dendrites in Distinct Medio-Lateral Territories

Finally, we asked what the significance might be of partitioning the neuropile into distinct dendritic domains. It is reasonable to suppose that muscles of similar position and orientation exert related functions, and so might operate in concert during locomotion. The myotopic segregation of motorneuron dendrites might therefore reflect differences in connectivity.

**Figure 10:**
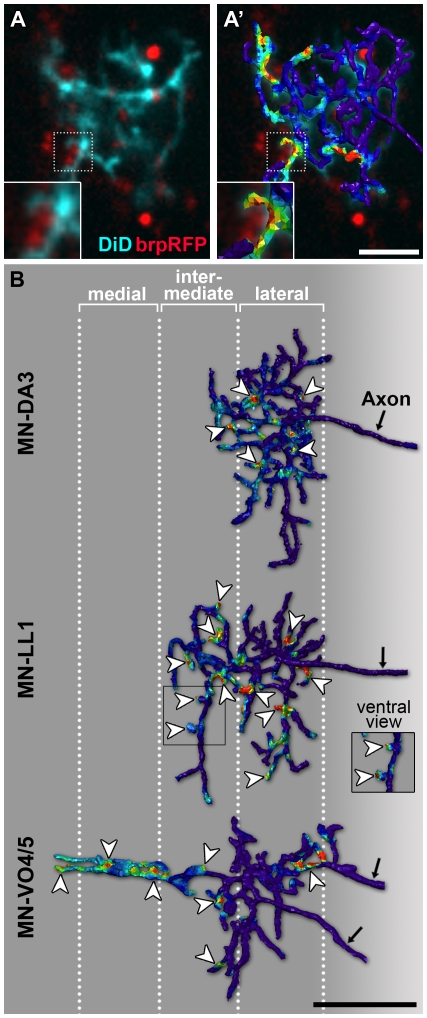
Medio-lateral distribution of excitatory presynaptic terminals on dendrites. (A) Single confocal section of a motorneuron in freshly hatched larvae (21 h AEL) retrogradely labelled with DiD (cyan) with cholinergic presynaptic sites visualised with *Cha-GAL4; UAS-brp-RFP* (red). Part of the arbor (stippled outline) is shown enlarged in the inset in the bottom left-hand corner. (A′) The same confocal section is shown as in (A) and superimposed is a digital 3-D reconstruction of the entire dendritic arbor. Relative probabilities of synaptic connections were mapped onto the reconstructed arbour; colours towards the red spectrum indicating high probabilities based on brp-RFP fluorescence signal intensity and distance to dendrites (<400 nm). The insets in (A and A′) show an enlarged view of brp-RFP puncta and DiD-labelled dendrite in close apposition. (B) Dorsal views (a ventral view of part of the MN-LL1 arbor is shown in the inset) of representative reconstructed dendritic trees from dye-labelled MN-DA3, MN-LL1, and MN-VO4/5 with the distribution of putative synaptic sites mapped onto these as illustrated in (A and A′). Putative synaptic contacts (arrowheads) can be found on medial, intermediate, and lateral dendritic branches. Anterior is up. Scale bar: 5 µm in (A, A′) (2.5 µm for insets); 10 µm in (B).

Since individual presynaptic partner neurons have not yet been identified, we sought to address this issue by asking whether motorneuron dendrites targeted to lateral, intermediate, or midline territories received presynaptic contacts. We visualised presynaptic active zones of cholinergic interneurons with *Cha^B19/7.4^-GAL4* driving *UAS-bruchpilot-mRFP* and labelled dendritic trees of MN-DA3 (*n* = 5), MN-LL1 (*n* = 6), and VO4/5-MN (*n* = 4) with DiD/DiO in newly hatched larvae (21 h AEL). Using custom-made image analysis software [25],[48],[50] we reconstructed the dendritic arbors of motorneurons and assayed for putative presynaptic specialisations on these (based on apposition within light microscopic resolution, approximately a 400 nm radius of the reconstructed dendrites). We find putative presynaptic sites on dendrites in the lateral, intermediate, and midline neuropile, though at present we cannot determine if these actually represent type-specific patterns of connectivity (Figure 10).

In the light of reports that suggest that different motorneurons in the *Drosophila* embryo receive different inputs [22], we interpret these observations as an indication that the segregation of motorneuron dendrites into distinct myotopic domains might be an underlying feature, or perhaps mechanism, of motorneuron class-specific patterns of connectivity.

## Discussion

Previously, we showed that motorneurons in the *Drosophila* embryo distribute their dendrites in distinct anterior to posterior domains in the neuropile, forming a central representation of target muscle positions in the periphery. The mechanisms required for the generation of this dendritic myotopic map remain elusive. In this study we have characterised dendritic myotopic organisation in a second dimension, with respect to the ventral midline, and we have identified the main molecular mechanism that underlies the formation of this dendritic neural map, namely the combinatorial action of the midline signalling systems Slit/Robo and Netrin/Frazzled.

### Myotopic Maps Might Organise Patterns of Connectivity in Motor Systems

Neural maps are manifestations of an organisational strategy commonly used by nervous systems to order synaptic connections. The view of these maps has been largely axonocentric and focused on sensory systems, though recent studies have challenged the notion of dendrites as a “passive” party in arranging the distribution of connections [13],[16],[51],[52]. Here, we have demonstrated that motorneuron dendrites generate a neural, myotopic map in a motor system and that this manifest regularity can form independently of its presynaptic partner terminals.

An essential feature of neural maps is the spatial segregation of synaptic connections. In the *Drosophila* embryonic nerve cord, there is some overlap between dendritic domains in the antero-posterior neuropile axis. Overlap of dendritic territories is also evident in the medio-lateral dimension, since all motorneurons have arborisations in the lateral neuropile, though distinctions arise by virtue of dendrites in additional intermediate and medial neuropile regions. The combination of myotopic mapping in both dimensions may serve to maximise the segregation between dendrites of different motorneuron groups. For example, the dendritic domain of motorneurons with dorsal targets differs from the territory innervated by ventrally projecting motorneurons in the antero-posterior location and the medio-lateral extent. Myotopic mapping in two dimensions could also provide a degree of flexibility that could facilitate wiring up in a combinatorial fashion. For instance, muscle LL1 lies at the interface between the dorsal and ventral muscle field; its motorneuron, MN-LL1, has one part of its dendritic arbor in the lateral domain that is characteristic for dorsally projecting motorneurons, while the other part of the dendritic tree innervates the intermediate neuropile precisely where ventrally projecting motorneurons put their dendrites.

Myotopic dendritic maps might constitute a general organisational principle in motor systems. In insects, a comparable system of organisation has now been demonstrated also for the adult motor system of *Drosophila* (see companion study by Brierley and colleagues [53] and [54]) and a degree of topographic organisation had previously been suggested for the dendrites of motorneurons that innervate the body wall muscles in the moth *Manduca sexta* [55]. In vertebrates too, there is evidence that different motor pools elaborate their dendrites in distinct regions of the spinal cord in chick, turtle, and mouse [4]–[6]. Moreover, elegant work in the mouse has shown that differences in dendritic territories correlate with and may determine the specificity of proprioceptive afferent inputs [6].

### The Myotopic Map Is Generated by the Combinatorial Action of the Midline Guidance Cue Receptors Robo and Frazzled

The neural map that we have characterised here is composed of three morphological classes of motorneurons with dendrites innervating either i) the lateral or ii) the lateral and intermediate or iii) the lateral, intermediate, and medial/midline neuropile (Figure 1).

We have shown that the motorneuron dendrites are targeted to these medio-lateral territories by the combinatorial, cell-autonomous actions of the midline guidance cue receptors Robo and Frazzled. The formation of dendritic territories by directed, targeted growth appears to be an important mechanism that may be more widespread than previously anticipated [56], though the underlying mechanisms may vary. Global patterning cues have been implicated in the vertebrate cortex (Sema3A [29]). In the zebrafish retina, live imaging has shown that retinal ganglion cells put their dendrites into specific strata of the inner plexiform layer, but the roles of guidance cues and interactions with partner (amacrine) cells have not yet been studied [13].

Slit/Robo and Netrin/Frazzled mediated gating of dendritic midline crossing has been previously documented in *Drosophila* embryos [28] and zebrafish [57]. Here, we demonstrate for the first time that dendrites are targeted to distinct medio-lateral territories by the combinatorial, opposing actions of Robo and Frazzled and that this is the main mechanism underlying the formation of the myotopic map. Strikingly, the same signalling pathways also regulate dendritic targeting of adult motorneurons in *Drosophila*, suggesting this to be a conserved mechanism (see companion paper [53]). Robo gates midline crossing of dendrites and in addition, at progressively higher signalling levels, restricts dendritic targeting to intermediate and lateral territories. Frazzled, on the other hand, is required for targeting dendrites towards the midline into intermediate and medial territories. Our data argue that Frazzled is expressed by representatives of all three motorneuron types (see also [58]). Recently, Yang and colleagues [59] showed that expression of *frazzled* leads to a concomitant transcriptional up-regulation of *comm*, thus linking Frazzled-mediated attraction to the midline with a decrease in Robo-mediated repulsion. While this has been demonstrated for midline crossing of axons in the *Drosophila* embryo, we found that, at least until 18.5 h AEL, expression of *UAS-frazzled* alone was not sufficient to induce midline crossing of dendrites in MN-LL1 and MN-DA3 (see Figure 6). It is conceivable that differences in expression levels and/or timing between *CQ-GAL4* used here and *egl-GAL4* used by Yang et al. might account for the differences in axonal and dendritic responses to *UAS-frazzled* expression. Moreover, the widespread expression of Frazzled in motorneurons and other cells in the CNS may point to additional functions, potentially synaptogenesis, as recently shown in *C. elegans* [60],[61].

Strikingly, neither synaptic excitatory activity nor the presynaptic (cholinergic) partner terminals seem to be necessary for the formation of the map. The map is already evident by 15 h AEL, before motorneurons receive synaptic inputs (Figure 2). It also forms in the absence of acetylcholine, the main (and at that stage probably exclusive) neurotransmitter to which motorneurons respond (Figure 3) [22]. Moreover, motorneuron dendrites innervate their characteristic dendritic domains when the cholinergic terminals have been displaced to outside the motor neuropile (Figure 4). However, interactions with presynaptic partners seem to contribute to its refinement. First, we find that dendritic mistargeting phenotypes show a greater degree of penetrance earlier (15 h AEL) than later (18.5 h AEL) in development (Figure S3). Secondly, when interactions with presynaptic partner terminals are reduced or absent, dendritic arbor size increases [25] and the distinction between dendritic territories is less evident than in controls (Figure 4H–4J). Fine-tuning of terminal arbors and sets of connections through contact and activity-dependent mechanisms is a well-established feature of neural maps in sensory systems (for review see [10],[62]) and our observations suggest that this may also apply to motor systems.

### Dendrite Morphology Is the Product of Separable Programmes for Growth and Branching and Targeting

The formation of the myotopic map is the product of dendritic targeting. It is therefore intimately linked with the question of how cell type–specific dendritic morphologies are specified. For instance, changing the balance between the Robo and Frazzled guidance receptors in motorneurons is sufficient to “convert” dendritic morphologies from one type to another (Figures 5 and 6). The importance of target territories for determining dendritic arbor morphology has recently been explored in a study of lobula plate tangential cells in the blowfly, where the distinguishing parameter between the dendritic trees of four functionally defined neurons were not growth or branching characteristics but the regions where neurons put their dendrites [63].

Because Slit/Robo and Netrin/Frazzled signalling have been reported to affect dendritic and axonal branching as well as axonal growth, respectively, we asked what the effect was on motorneuron dendrites of altered Robo and Frazzled levels [33],[42]–[47]. We find that in the wild-type different motorneurons generate characteristically different amounts of dendritic length and numbers of branch points (MN-DA1/aCC and MN-VO2/RP1 [25], RP2 [34], MN-DA3 and MN-LL1, this study). In the *Drosophila* embryo and larva, Slit/Robo interactions have been suggested to promote the formation of dendrites and/or branching events [44],[45], similar to what had previously been shown for cultured vertebrate neurons [47]. Our data on embryonic motorneurons are not compatible with this interpretation. First, when altering the levels of Robo (or Frazzled) in individual motorneurons and mistargeting their dendrites, we could not detect statistically significant changes in total dendritic length or number of branch points. Instead, for MN-DA3 and MN-LL1, we observed that dendritic arbors respond to changes in the expression levels of midline cue receptors by altering the amount of dendritic length distributed to the medial, intermediate, and lateral neuropile (Figures 8 and 9). Secondly, in nerve cords entirely mutant for the Slit receptor Robo we see an increase in dendrite branching at the midline (Figure S4). Our observations suggest that for *Drosophila* motorneurons Slit/Robo interactions negatively regulate the establishment and branching of dendrites and thus specify dendritic target territories by defining “exclusion” zones in the neuropile. The quantitative data from this and a companion study [53] suggest that dendritic morphology is the product of two intrinsic, genetically separable programmes: one that specifies the total dendritic length to be generated and the frequency of branching; the other implements the distribution of these dendrites in the target territory, presumably by locally modulating rates of extension, stabilisation, and retraction of branches in response to extrinsic signals. Observations from a previous study [34] and other systems, e.g., insect sensory neurons [64] and vertebrate cortical neurons [65], are compatible with this model.

### Specification of Connectivity by Global Patterning Cues

The question of how neural circuits are generated remains at the heart of developmental neurobiology. At one extreme, one could envisage that every synapse was genetically specified, the product of an exquisitely choreographed sequence of cell-cell interactions. At the other extreme, neural networks might assemble through random cell-cell interactions and feedback processes enabling functional validation. The latter view supposes that neurons inherently generate polarised processes, have a high propensity to form synapses, and arrive at a favourable activity state through homeostatic mechanisms. Current evidence suggests that, at least for most systems, circuits form by a combination of genetic specification and the capacity to self-organise (for reviews see [10],[62],[66]).

In this study we have demonstrated that the postsynaptic structures of motorneurons, the dendrites, form a neural map. We have also shown that dendrites are closely apposed to cholinergic presynaptic specialisations in their target territories (Figure 10), suggesting that the segregation of dendrites may be a mechanism that facilitates the formation of specific sets of connections. Strikingly, this map of postsynaptic dendrites appears to be “hard-wired” in that it can form independently of its presynaptic partners and it is generated in response to a third party, the midline guidance cues Slit and Netrin (see also companion paper [53]). A comparable example is the *Drosophila* antennal lobe, where projection neurons form a neural map independently of their presynaptic olfactory receptor neurons, though in this sensory system the nature and source of the cue(s) remain to be determined [15],[16]. With this study we complement previous work that demonstrated the positioning of presynaptic axon terminals by midline cues, also independently of their synaptic partners [17],[18]. Together, these results suggest that global patterning cues set up the functional architecture of the nervous system by independently directing pre- and postsynaptic partner terminals towards common “meeting” areas.

Clearly, such global guidance systems deliver a relatively coarse level of specificity and there is ample evidence for the existence of codes of cell-adhesion molecules and local receptor-ligand interactions capable of conferring a high degree of synaptic specificity [67]–[75]. Therefore, one has to ask what the contribution is of global partitioning systems in establishing patterns of connections that lead to a functional neural network. A recent study in the *Xenopus* tadpole spinal cord has addressed this issue. Conducting patch clamp recordings from pairs of neurons, Li and colleagues [76] found that the actual pattern of connections in the motor circuit reveals a remarkable lack of specificity. Furthermore, the segregation of axons and dendrites into a few broad domains appears to be sufficient to generate the connections that do form and to enable the emergence of a functional network [76]. The implication is that neurons might be intrinsically promiscuous and targeting nerve terminals to distinct territories by global patterning cues, as we have shown here, is important to restrict this synaptogenic potential and thereby confer a degree of specificity that is necessary for the emergence of network function.

## Material and Methods

### Fly Stocks

The following fly stocks were used: Oregon-R, *Fasciclin2-GFP* on X (always used in a heterozygous condition in females [*w^−^*, *Fasciclin2-GFP/w^−^*] [77]), amorphic *fra^3^/fra^4^* [78], amorphic *netAB^Δ^* [40], *UAS-fra^myc^* on III [78], amorphic *robo^1^/robo^GA1112^* [79], *UAS-robo* two insertions 2B, 3D on III [79], *Cha^B19/7.4^-GAL4* on II [80], *UAS-commissureless* on X [36], *UAS-Robo^ex^Fra^in^-myc* and *UAS-Fra^ex^Robo^in^-myc* [31], *UAS-bruchpilot-mRFP* on III (generously provided by S. Mertel and S. Sigrist), c*ha^l13^* [30], and *UAS-myr-mRFP1* (generated by Henry Cheng, obtained from the Bloomington Stock Center). Lethal mutations/insertions were kept over *FM7*, *CyO*, and *TM3* balancer chromosomes that are additionally marked with *Kr-GAL4*, *UAS-GFP* [81]. Selective expression in MN-DA3, MN-LL1, and MN-VO4–6 was achieved using *CQ-GAL4* with insertions on chromosomes II and III. This line expresses in the five CQ/U-motorneurons MN-DO1, MN-DO2, MN-DA2, MN-DA3, and MN-LL1 [3] as well as MN-VO4–6 in approximately 30% of half segments, always confirmed in expression experiments by the presence of an additional reporter, *UAS-bruchpilot-mRFP*, at respective NMJs. Rarely, up to eight cells per half segment can be seen expressing with *CQ-GAL4*, indicating potential expression in one or two interneurons, though we have no evidence of these having terminations in the motor neuropile.

### Genotypes of Embryos Used for Analysis:

*robo* mutant:

 *w* ^−^, *Fas2GFP/w* ^−^; *robo^1^/robo^GA1112^*

Down-regulation of Robo:

 *w* ^−^, *Fas2GFP/w* ^−^, *UAS-comm*; *CQ-GAL4/+*; *CQ-GAL4/+*

Robo expression:

 w^−^, *Fas2GFP/w* ^−^; *CQ-GAL4/+; CQ-GAL4, UAS-brpRFP/UAS-robo2B, 3D* (for MN-VO4–6)

 *w* ^−^, *Fas2GFP/w* ^−^; *CQ-GAL4/+; CQ-GAL4; UAS- robo2B, 3D* (for MN-LL1 and MN-DA3)

*robo* mutant with cell-autonomous rescue:

 *w* ^−^, *Fas2GFP/w* ^−^; *robo^1^/robo^GA1112^*; *CQ-GAL4*; *UAS-robo2B*, *3D*

*frazzled* mutant:

 *w* ^−^, *Fas2GFP/w* ^−^; *fra^3^/fra^4^*, *CQ-GAL4*; *CQ-GAL4/+*

Frazzled expression:

 *w_2_, Fas2GFP/w_2_; CQ-GAL4/+; CQ-GAL4 / UAS-fra-myc*

*frazzled* mutant with cell-autonomous rescue of *frazzled*:

 *w* ^−^, *Fas2GFP/w* ^−^; *fra^4^*, *CQ-GAL4/fra^3^*; *CQ-GAL4*, *UAS-brpRFP/UAS-fra-myc* (for MN-VO4–6)

 *w* ^−^, *Fas2GFP/w* ^−^; *fra^4^*, *CQ-GAL4/fra^3^*; *CQ-GAL4/UAS-fra-myc* (for MN-LL1)

Down-regulation of Robo in a *frazzled* mutant:

 *w* ^−^, *Fas2GFP/w* ^−^, *UAS-comm*; *fra^4^*, *CQ-GAL4/fra^3^*; *CQ-GAL4/+*

Down-regulation of Robo in a *frazzled* mutant with cell-autonomous rescue of *frazzled*:

 *w* ^−^, *Fas2GFP/w* ^−^, *UAS-comm*; *fra^4^*, *CQ-GAL4/fra^3^*; *CQ-GAL4/UAS-fra-myc*

*netrin* mutant:

 *w* ^−^, *Fas2GFP*, *netAB^Δ^/y*

Labelling of cholinergic presynaptic sites:

 *w* ^−^; *Cha^B19/7.4^-GAL4/+*; *UAS-brpRFP/*+ or *UAS-brpRFP*

Labelling and shifting of cholinergic terminals:

 *w* ^−^, *Fas2GFP/w* ^−^; *Cha^B19/7.4^-GAL4, UAS-myr-mRFP/+; +/+*

 *w* ^−^, *Fas2GFP/w* ^−^; *Cha^B19/7.4^-GAL4, UAS-myr-mRFP/UAS-Fra^ex^-Robo^in^-myc; +/+*

 *w* ^−^, *Fas2GFP/w* ^−^; *Cha^B19/7.4^-GAL4, UAS-myr-mRFP/UAS-Robo^ex^-Fra^in^-myc; +/+*

### Cell Labelling

Embryos 15 h AEL were dissected as described in [19], though without collagenase treatment; embryos 18.5 h AEL (onset of trachea filling) were dissected as described in [21]. Embryos were then fixed with 3.7% formaldehyde in saline for 2.5 min and rinsed. Retrograde labellings were done as described by [19], and in addition neuromuscular junctions were visualised by FITC-conjugated anti-horseradish peroxidase incubation for ∼3–6 min (Jackson ImmunoResearch, West Grove, PA, United States; 1∶50 dilution in saline), followed by saline washes. Neuro-DiO (Biotium), DiD, and DiI (Molecular Probes, Eugene, OR, United States) were used at 2 mg/ml, 2 mg/ml, and 4 mg/ml, respectively, dissolved in vegetable oil. Anterograde Lucifer Yellow (Invitrogen) labellings were done as in [18].

### Confocal Imaging and Data Acquisition

Labelled neurons were imaged with a Yokagawa CSU-22 confocal field scanner mounted on an Olympus BX51WI Spinning Disc microscope, using a 63×/1.2 NA (Olympus) water immersion objective. Image z-stacks were acquired using MetaMorph software (Molecular Devices) and processed using ImageJ 1.39 s software (U.S. National Institutes of Health, Bethesda, MD, USA, http://rsb.info.nih.gov/ij/). Cumulative dendrite plots: z-projections of dendritic trees were scaled and aligned isometrically onto a common reference grid with Photoshop CS2 (Adobe Systems, San Jose, CA, USA), using the position of the motorneuron axon in one channel as the antero-posterior and the outer and inner Fasciclin2-positive axon tracts as medio-lateral reference points. Silhouettes (intensity information was discarded) of dendritic trees of each experimental condition were summed using ImageJ.

### Reconstruction of Dendritic Trees

Dual channel confocal image stacks were generated (z-step size: 300 nm) of Neuro-DiO labelled dendrites and a presynaptic marker expressed in cholinergic neurons (*w* ^−^; *Cha^B19/7.4^-GAL4/+; UAS-bruchpilot-mRFP/*+ or *w* ^−^; *Cha^B19/7.4^-GAL4/+; UAS-bruchpilot-mRFP/UAS-bruchpilot-mRFP*). Dendrites were reconstructed using a custom-made module [48]–[50] for AMIRA software (version 4.1). Relative probabilities of synaptic contact on reconstructed dendrites were calculated based on both the distance and signal intensity of presynaptic mRFP-puncta, and represented by a colour code, ranging from blue (indicating a relatively low probability of contact) to red (<400 nm distance, indicating a relatively high probability of synaptic contact).

### Statistical Analysis

Geometrical data from dendritic “skeleton trees” were exported from AMIRA as csv-files, analysed, and plotted using “R project” (R Foundation for Statistical Computing, Vienna, Austria, 2005. http://R-project.org). Data were analysed statistically using the Shapiro-Wilk test to assess for normality followed by a Student's *t* test or a Wilcoxon rank-sum test as appropriate.

## Supporting Information

Figure S1 **Identification of the RP1 and RP4 motorneuron target muscles.** The dorsally located cell bodies of the RP1 and RP4 motorneurons were filled intracellularly with Lucifer Yellow (green) to identify the target muscles in the abdomen of the embryo at 18.5 h AEL. F-actin in muscles was stained using Phalloidin (red). One representative image of the central and peripheral arbors is shown for each motorneuron (A–B′). Cell body position, dendritic morphology, and target muscle identity strongly correlate (see table, note that not all cell body positions could be unambiguously classified). The RP1 motorneuron can thus be identified by its posterior intersegmental dendrite ([A] curved arrow) and its VO2 target muscle ([A′] arrow head indicates axon terminal). RP4 does not generate an intersegmental dendrite and innervates muscle VO1 in the periphery ([B′] arrow head). In addition, the RP1 cell body ([A] asterisk) tends to lie just across the midline on the contralateral side of the muscle that it innervates (dotted line) whereas the RP4 soma ([B] asterisk) is usually situated next to RP1, one cell diameter away from the midline, as originally defined by Halpern and colleagues [87]. (C, C′) Retrograde DiD-fills of the RP5 motorneuron from muscle VL2 reveal that at 21 h AEL the RP5 axon arborises over most ventral internal muscles except muscles VO3 and VO6. Note that the VL2-specific motorneuron (MN-VL2) was also labelled. (D, D′) The motorneuron DiD labelled from muscle VO6 does also form boutons in the VO4/VO5 muscle cleft at 18.5 h AEL ([D′] arrowhead). This motorneuron is therefore termed “MN-VO4–6.” The inset in (D′) shows a higher magnification of the MN-VO4–6 terminal. Dotted line: CNS midline. Straight arrows: motorneuron axons. Scale bars (except for inset in [D′]): 20 µm.
(3.56 MB TIF)

Figure S2 **Central morphology of identified type Ib-motorneurons innervating the internal muscle field.** Panels show z-projections of 16 DiI/DiD-filled identified motorneurons (two for each) targeting the indicated muscles in abdominal segments 2–6. Cell body and target muscle positions of the motorneurons are depicted in the diagram (left, CNS cross section; right, internal muscle field). Motorneurons and muscles are colour-coded according to the lateral-to-medial extent of the corresponding dendritic territories in the CNS: magenta, lateral; green, lateral and intermediate; yellow, lateral, intermediate, and medial/midline. Anterior is up. Scale bar: 20 µm.
(7.05 MB TIF)

Figure S3 **Robo and Frazzled signalling in motorneurons set up dendritic medio-lateral territories by 15 h AEL.** Dendritic trees of DiI/DiD-filled LL1 and DA3 motorneurons at 15 h AEL (before synapses become functional at 16 h AEL [21]) in *fra-* or *robo-*manipulated genetic backgrounds (mutant alleles or UAS-transgenes selectively expressed in MN-LL1 and MN-DA3 with *CQ-GAL4* are indicated). Frazzled expression in the motorneuron is necessary and sufficient for dendritic targeting to the intermediate neuropile (located between the intermediate and medial Fasciclin2-positive axon tract; compare [A, D, and F]). Loss or down-regulation of Robo (by *UAS-comm* expression) leads to ectopic growth of dendrites to the midline (G, H, I). Phenotypes are consistent with an early role of the receptors for medio-lateral dendritic patterning: MN-LL1 adopts a MN-DA3-like morphology when *fra* is absent (compare [D] with [C and D]) while MN-DA3 produces a prominent intermediate dendritic arbor normally characteristic for MN-LL1 upon overexpression of *UAS-fra* (compare [F] with [A]). The penetrance of phenotypes of loss and overexpression of Frazzled is greater at this earlier stage than at 18.5 h AEL: in *fra^3^/fra^4^* mutant embryos 88% of MN-LL1 fail to put dendrites into the intermediate territory at 15 h AEL (*n* = 15) as compared to 64% at 18.5 h AEL (*n* = 18); overexpression of Frazzled in MN-DA3 leads to ectopic dendritic elaboration in the intermediate neuropile in 76% of cases at 15 h AEL (*n* = 17) as compared to 50% at 18.5 h AEL (*n* = 20). Dotted line: CNS midline. Scale bar: 5 µm.
(3.00 MB TIF)

Figure S4 **Dendritic morphologies of motorneurons in** ***robo*** **-mutant embryos.** z-projection views of dendritic trees are shown for four motorneurons (three for each MN-VO4/5, MN-DA1 [aCC], MN-LL1, and MN-DA3) to compare the size and branching of dendritic arbors between wild-type and *robo^1^/robo^GA1112^* mutant conditions at 18.5 h AEL (A–D). MN-VO4/5, MN-LL1, and MN-DA3 (A, C, D) all appear to have increased dendritic length and branch point numbers in embryos entirely mutant for *robo*. These parameters seem to be least affected in MN-DA1 (aCC) (B). For MN-LL1 and MN-DA3 length and tip numbers were precisely quantified from reconstructed dendritic trees ([C′] MN-LL1, *n* = 5; [D′] MN-DA3, *n* = 9): MN-LL1 dendrites in *robo^1^/robo^GA1112^* show a statistically significant increase in both parameters compared to wild-type controls. Although MN-DA3 dendritic arbors show a similar trend, these changes are not statistically significant. Student's *t* test and Wilcoxon test were used for statistical analysis as appropriate. Arrowheads indicate the position of the CNS midline. Anterior is up. Scale bar: 20 µm.
(1.17 MB TIF)

Table S1 **Neuromuscular connectivity of the internal abdominal muscle field.** The table lists the names of glutamatergic type-Ib and type-Is motorneurons and their targets within the internal muscle field. Peptidergic/modulatory motorneurons also innervating these muscles, such as the V-neuron (innervates muscle VL1 with type-III boutons) or the VUM neurons (type-II boutons, one innervating the ventral, another the set of dorsal internal muscles) have not been listed. For muscle VL1 we have not been able to identify a unique type-Ib motorneuron at 15 h and 18 h AEL, only innervation by RP5, the common exciter, a VUM neuron and the V-neuron. Our observations are largely in agreement with recent studies^c^. Corroborative references are provided for those motorneuron-muscle pairs where there had previously been inconsistencies on their connectivity either among these studies or with this work. References: a [84], b [85], c [3], d [86].
(0.02 MB XLS)

## Abbreviations

AEL: after egg laying
brp: bruchpilot
Cha: choline acetyltransferase
CNS: central nervous system
comm: commissureless
egl: eagle
fra: frazzled
GFP: green fluorescent protein
RFP: red fluorescent protein
robo: roundabout
