# Supplementary figures and images for: Midline Signalling Systems Direct the Formation of a Neural Map by Dendritic Targeting in the Drosophila Motor System
Source: PLoS Biol. 2009 Sep 22;7(9):e1000200. doi: 10.1371/journal.pbio.1000200 (PMC2736389; doi:10.1371/journal.pbio.1000200)

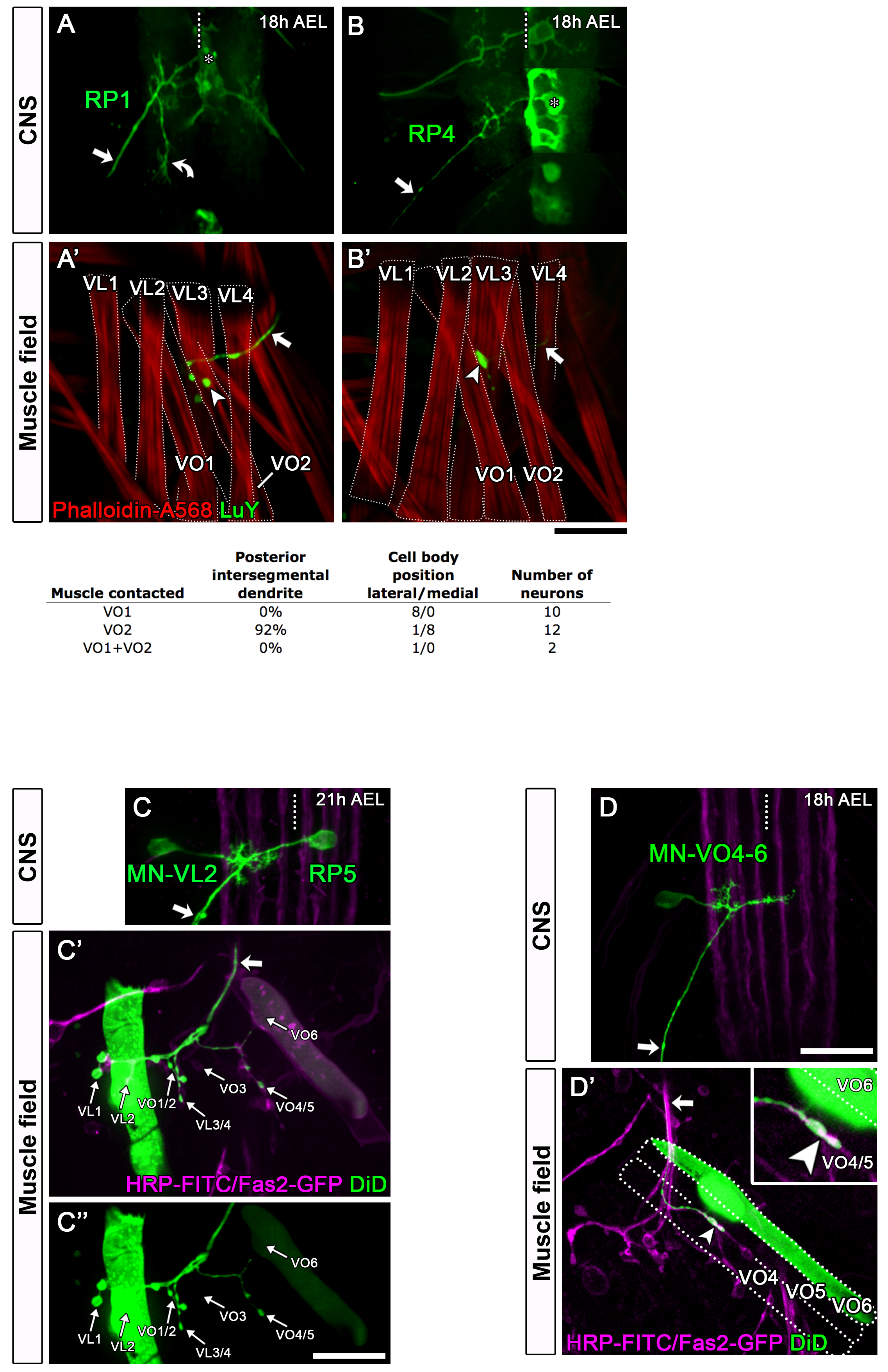

Supplement: Figure S1 — Identification of the RP1 and RP4 motorneuron target muscles. The dorsally located cell bodies of the RP1 and RP4 motorneurons were filled intracellularly with Lucifer Yellow (green) to identify the target muscles in the abdomen of the embryo at 18.5 h AEL. F-actin in muscles was stained using Phalloidin (red). One representative image of the central and peripheral arbors is shown for each motorneuron (A–B′). Cell body position, dendritic morphology, and target muscle identity strongly correlate (see table, note that not all cell body positions could be unambiguously classified). The RP1 motorneuron can thus be identified by its posterior intersegmental dendrite ([A] curved arrow) and its VO2 target muscle ([A′] arrow head indicates axon terminal). RP4 does not generate an intersegmental dendrite and innervates muscle VO1 in the periphery ([B′] arrow head). In addition, the RP1 cell body ([A] asterisk) tends to lie just across the midline on the contralateral side of the muscle that it innervates (dotted line) whereas the RP4 soma ([B] asterisk) is usually situated next to RP1, one cell diameter away from the midline, as originally defined by Halpern and colleagues [87]. (C, C′) Retrograde DiD-fills of the RP5 motorneuron from muscle VL2 reveal that at 21 h AEL the RP5 axon arborises over most ventral internal muscles except muscles VO3 and VO6. Note that the VL2-specific motorneuron (MN-VL2) was also labelled. (D, D′) The motorneuron DiD labelled from muscle VO6 does also form boutons in the VO4/VO5 muscle cleft at 18.5 h AEL ([D′] arrowhead). This motorneuron is therefore termed “MN-VO4–6.” The inset in (D′) shows a higher magnification of the MN-VO4–6 terminal. Dotted line: CNS midline. Straight arrows: motorneuron axons. Scale bars (except for inset in [D′]): 20 µm. (3.56 MB TIF) [file pbio.1000200.s001.tif]

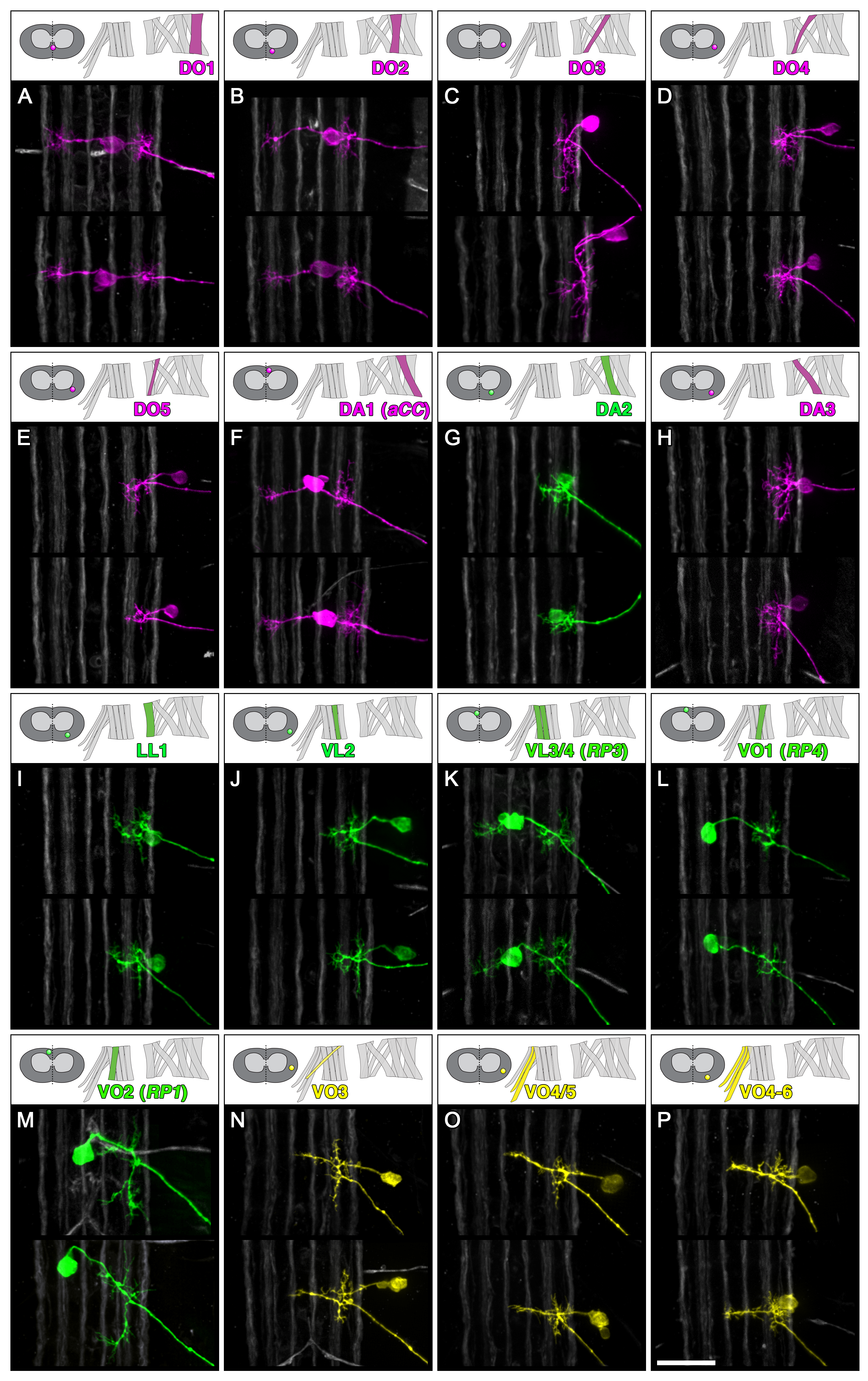

Supplement: Figure S2 — Central morphology of identified type Ib-motorneurons innervating the internal muscle field. Panels show z-projections of 16 DiI/DiD-filled identified motorneurons (two for each) targeting the indicated muscles in abdominal segments 2–6. Cell body and target muscle positions of the motorneurons are depicted in the diagram (left, CNS cross section; right, internal muscle field). Motorneurons and muscles are colour-coded according to the lateral-to-medial extent of the corresponding dendritic territories in the CNS: magenta, lateral; green, lateral and intermediate; yellow, lateral, intermediate, and medial/midline. Anterior is up. Scale bar: 20 µm. (7.05 MB TIF) [file pbio.1000200.s002.tif]

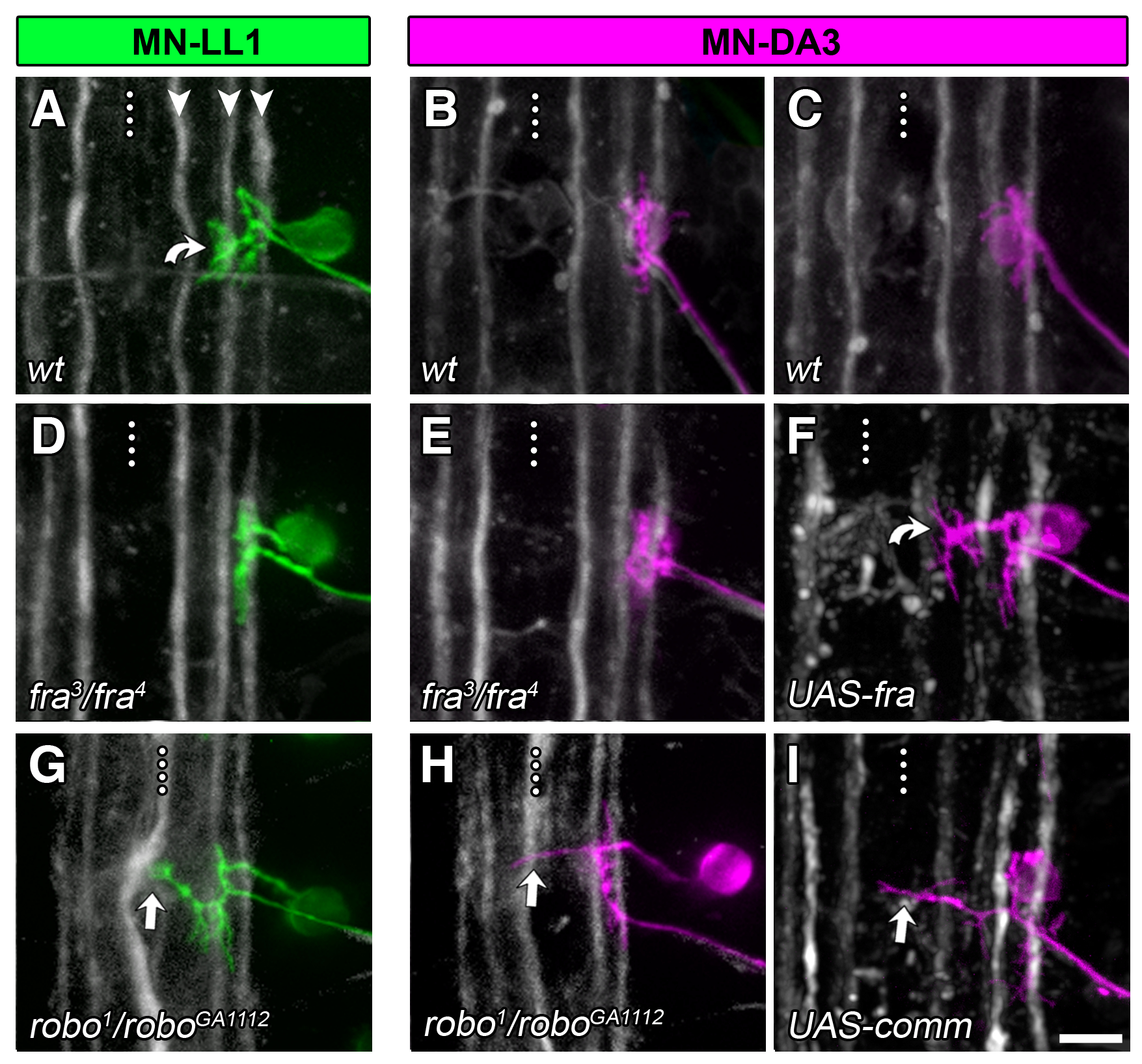

Supplement: Figure S3 — Robo and Frazzled signalling in motorneurons set up dendritic medio-lateral territories by 15 h AEL. Dendritic trees of DiI/DiD-filled LL1 and DA3 motorneurons at 15 h AEL (before synapses become functional at 16 h AEL [21]) in fra- or robo-manipulated genetic backgrounds (mutant alleles or UAS-transgenes selectively expressed in MN-LL1 and MN-DA3 with CQ-GAL4 are indicated). Frazzled expression in the motorneuron is necessary and sufficient for dendritic targeting to the intermediate neuropile (located between the intermediate and medial Fasciclin2-positive axon tract; compare [A, D, and F]). Loss or down-regulation of Robo (by UAS-comm expression) leads to ectopic growth of dendrites to the midline (G, H, I). Phenotypes are consistent with an early role of the receptors for medio-lateral dendritic patterning: MN-LL1 adopts a MN-DA3-like morphology when fra is absent (compare [D] with [C and D]) while MN-DA3 produces a prominent intermediate dendritic arbor normally characteristic for MN-LL1 upon overexpression of UAS-fra (compare [F] with [A]). The penetrance of phenotypes of loss and overexpression of Frazzled is greater at this earlier stage than at 18.5 h AEL: in fra3/fra4 mutant embryos 88% of MN-LL1 fail to put dendrites into the intermediate territory at 15 h AEL (n = 15) as compared to 64% at 18.5 h AEL (n = 18); overexpression of Frazzled in MN-DA3 leads to ectopic dendritic elaboration in the intermediate neuropile in 76% of cases at 15 h AEL (n = 17) as compared to 50% at 18.5 h AEL (n = 20). Dotted line: CNS midline. Scale bar: 5 µm. (3.00 MB TIF) [file pbio.1000200.s003.tif]

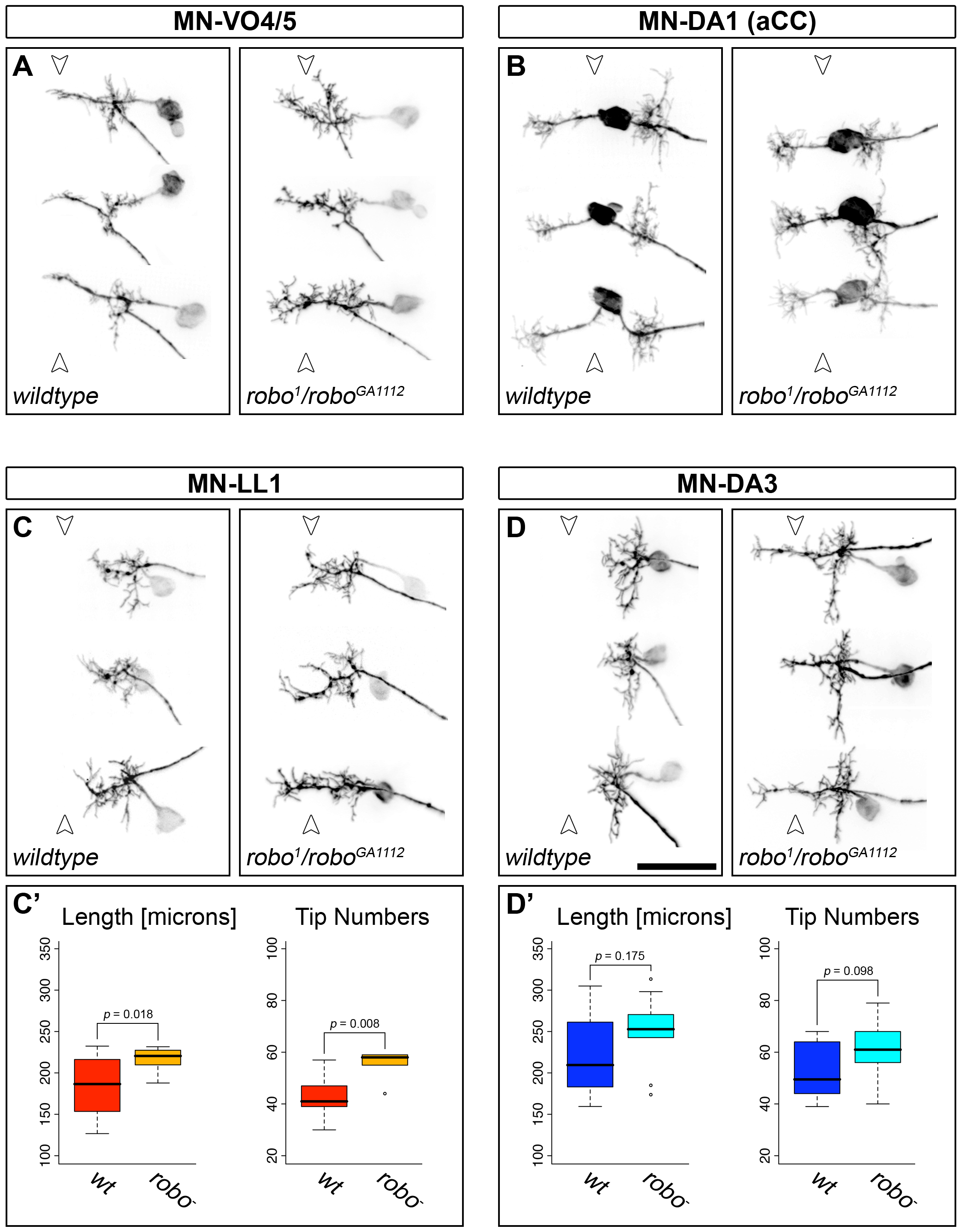

Supplement: Figure S4 — Dendritic morphologies of motorneurons in robo -mutant embryos. z-projection views of dendritic trees are shown for four motorneurons (three for each MN-VO4/5, MN-DA1 [aCC], MN-LL1, and MN-DA3) to compare the size and branching of dendritic arbors between wild-type and robo1/roboGA1112 mutant conditions at 18.5 h AEL (A–D). MN-VO4/5, MN-LL1, and MN-DA3 (A, C, D) all appear to have increased dendritic length and branch point numbers in embryos entirely mutant for robo. These parameters seem to be least affected in MN-DA1 (aCC) (B). For MN-LL1 and MN-DA3 length and tip numbers were precisely quantified from reconstructed dendritic trees ([C′] MN-LL1, n = 5; [D′] MN-DA3, n = 9): MN-LL1 dendrites in robo1/roboGA1112 show a statistically significant increase in both parameters compared to wild-type controls. Although MN-DA3 dendritic arbors show a similar trend, these changes are not statistically significant. Student's t test and Wilcoxon test were used for statistical analysis as appropriate. Arrowheads indicate the position of the CNS midline. Anterior is up. Scale bar: 20 µm. (1.17 MB TIF) [file pbio.1000200.s004.tif]
